# Metagenomic Profiling of Microbial Composition and Antibiotic Resistance Determinants in Puget Sound

**DOI:** 10.1371/journal.pone.0048000

**Published:** 2012-10-29

**Authors:** Jesse A. Port, James C. Wallace, William C. Griffith, Elaine M. Faustman

**Affiliations:** 1 Department of Environmental and Occupational Health Sciences, School of Public Health, University of Washington, Seattle, Washington, United States of America; 2 Institute for Risk Analysis and Risk Communication, School of Public Health, University of Washington, Seattle, Washington, United States of America; J. Craig Venter Institute, United States of America

## Abstract

Human-health relevant impacts on marine ecosystems are increasing on both spatial and temporal scales. Traditional indicators for environmental health monitoring and microbial risk assessment have relied primarily on single species analyses and have provided only limited spatial and temporal information. More high-throughput, broad-scale approaches to evaluate these impacts are therefore needed to provide a platform for informing public health. This study uses shotgun metagenomics to survey the taxonomic composition and antibiotic resistance determinant content of surface water bacterial communities in the Puget Sound estuary. Metagenomic DNA was collected at six sites in Puget Sound in addition to one wastewater treatment plant (WWTP) that discharges into the Sound and pyrosequenced. A total of ∼550 Mbp (1.4 million reads) were obtained, 22 Mbp of which could be assembled into contigs. While the taxonomic and resistance determinant profiles across the open Sound samples were similar, unique signatures were identified when comparing these profiles across the open Sound, a nearshore marina and WWTP effluent. The open Sound was dominated by α-Proteobacteria (in particular *Rhodobacterales sp.*), γ-Proteobacteria and Bacteroidetes while the marina and effluent had increased abundances of Actinobacteria, β-Proteobacteria and Firmicutes. There was a significant increase in the antibiotic resistance gene signal from the open Sound to marina to WWTP effluent, suggestive of a potential link to human impacts. Mobile genetic elements associated with environmental and pathogenic bacteria were also differentially abundant across the samples. This study is the first comparative metagenomic survey of Puget Sound and provides baseline data for further assessments of community composition and antibiotic resistance determinants in the environment using next generation sequencing technologies. In addition, these genomic signals of potential human impact can be used to guide initial public health monitoring as well as more targeted and functionally-based investigations.

## Introduction

Coastal ecosystems continue to be subjected to increasing human pressures. Over 40% of the global population currently lives within 100 km of coastlines, and this percentage continues to increase [1]. Human impacts have led to significant degradation in marine environments in the form of habitat loss, eutrophication, hypoxia, organic and inorganic pollution, invasive species, pathogen spread and ocean acidification, among others [2]. Based on a spatial analysis of 17 anthropogenic drivers, it has been estimated that over 40% of the world’s oceans have medium to high impacts associated with human activities [3]. These impacts subsequently now pose risks to human health in the form of bacterial and viral pathogens, harmful algal blooms, contaminated seafood and decreased well-being [4], [5], [6], [7]. There is thus considerable interest in investigating the distribution and magnitude of these impacts in marine environments, including nearshore, coastal and open ocean locations.

Assessing human impacts on such a global scale is a challenge, and current approaches to environmental monitoring are not well suited for large-scale spatial and temporal analyses. This is changing with advancements in the field of environmental genomics. Metagenomics, in tandem with next generation sequencing, now provides a technical means by which to monitor environmental microbial communities in a high-throughput manner. Metagenomics refers to the sequencing of DNA directly from environmental samples (i.e. metagenomes), and as such simultaneously provides access to the genetic information from mixed environmental microbial communities [8]. With this approach, taxonomic and functional microbial diversity can be profiled and community change subsequently monitored over space and time in response to environmental or anthropogenic impacts relevant to human health [9]. Microbial community composition has been shown to change in differentially impacted coastal environments [10], [11], [12] and other anthropogenically impacted environments [8], [13], [14], [15] and thus provides a potential indicator of community stressors and stress response.

Despite the potential role aquatic ecosystems may play in harboring and disseminating antibiotic resistance, little is known regarding the distribution of antibiotic resistance determinants within marine microbial communities. Antibiotic resistance continues to be a serious public health concern, as the overuse and misuse of antibiotics has led to the selection of antibiotic resistance genes in bacterial populations and has thus compromised our ability to treat bacterial infections. Coastal environments are subject to a multitude of anthropogenic impacts such as sewage, hospital waste, agricultural runoff and aquaculture that increase the prevalence of antibiotic resistance determinants in microbial communities, and thus these ecosystems have the potential to be important sources and sinks for antibiotic resistant bacteria and genes and may contribute to the dissemination of resistance determinants across organisms [16], [17]. In fact, many resistance genes found in pathogenic bacteria have evolved or are sourced from resistance genes found in environmental microbial communities [18]. Clinical resistance in many cases is therefore the result of horizontal transfer of resistance genes via mobile genetic elements from ecologically and taxonomically distant bacteria [19], and this gene transfer has been shown to occur across a wide range of bacterial species, including pathogens to non-pathogens and vice versa [20], [21], [22]. Furthermore, the marine environment in particular has been shown to have a high rate of horizontal gene transfer [23].

The majority of studies assessing the prevalence and distribution of antibiotic resistance genes in natural environments to this point have focused on specific known resistance genes using PCR-based techniques [24], [25], [26]. While these techniques provide resistance profiles for specific organisms or targeted genes, they are limited for ecosystem level applications because of these gene-by-gene and single species approaches. Thus there is limited knowledge regarding the global prevalence and distribution of antibiotic resistance genes and other resistance determinants including mobile genetic elements in natural microbial communities, and this data gap has been exacerbated by our inability to access the genomic information for unculturable bacteria, which represent >99% of bacteria in the environment [27]. Metagenomics now offers a culture-independent and high-throughput approach to investigate antibiotic resistance determinants in the environment at the microbial community-level, and a number of studies have already successfully employed this approach [28], [29], [30], [31], [32], [33], [34].

**Figure 1:**
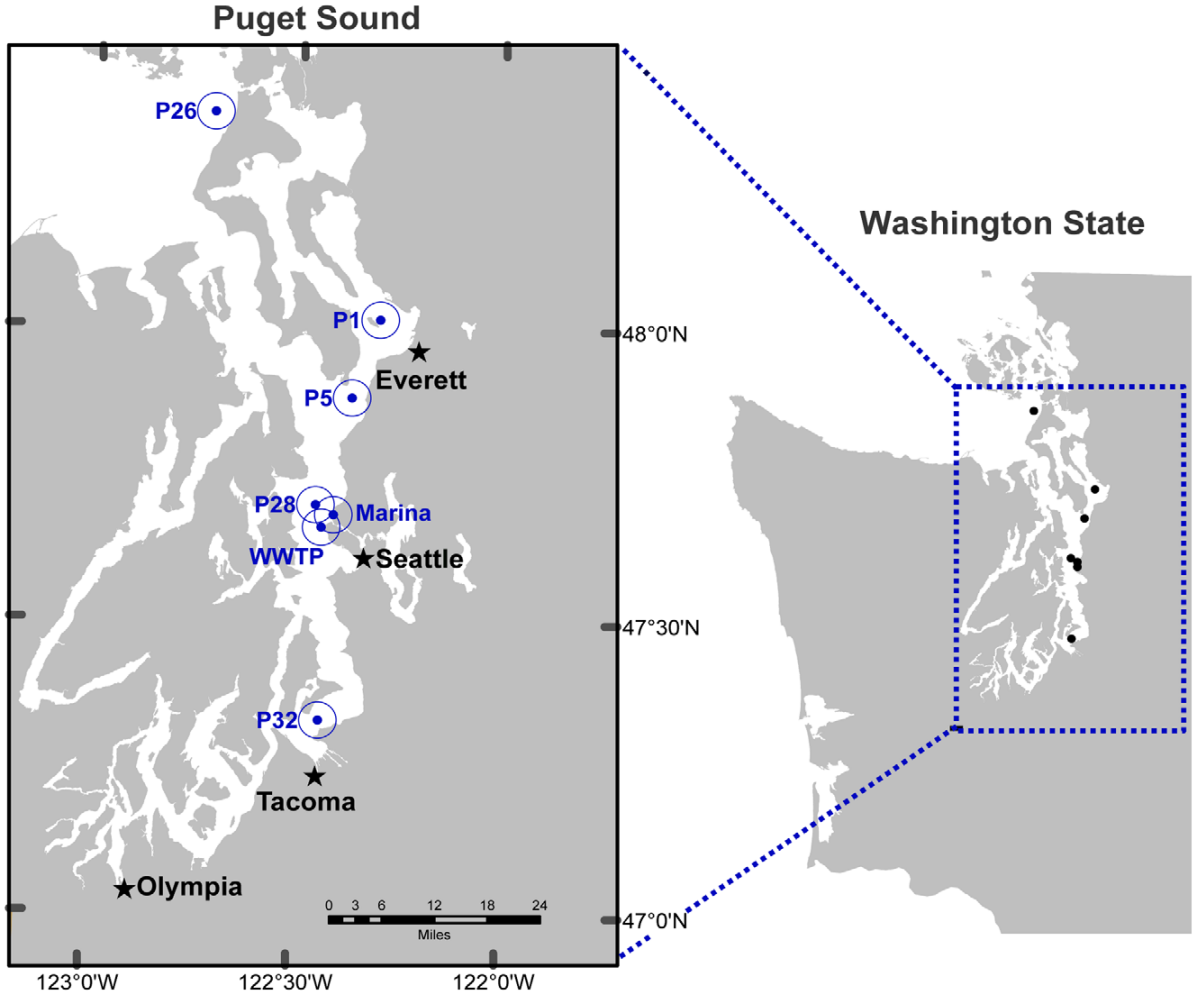
Locations of sampling sites in Puget Sound. Refer to Table 1 for the geographic coordinates of the sampling stations. WWTP, Wastewater treatment plant.

This study uses a metagenomic approach in combination with next generation sequencing to evaluate potential differences in community composition and antibiotic resistance gene signals across a natural ecosystem using the case study of Puget Sound, Washington. Puget Sound is a partially mixed fjord-like estuary that is connected to the Pacific Ocean via the Strait of Juan de Fuca and that receives the majority of its freshwater input from rivers emptying into the Whidbey Basin region (Figure 1) [35]. There are 96 publicly owned wastewater treatment plants (WWTP) in the Puget Sound Basin that serve over 3.5 million people from urban population centers such as Seattle, Tacoma, Olympia and Everett and process over 124 million gallons of sewage per day [36]. Antibiotics, heavy metals and other pollutants also enter the Sound through agricultural and urban runoff. At the same time, Puget Sound supports one of the largest shellfish industries in the country, with over 100 commercial growing areas covering 900 miles of shoreline [37]. The potential for these anthropogenic impacts to alter microbial community and resistance determinant composition in Puget Sound is uncertain, and baseline data is needed in order to spatially and temporally monitor these potentially health relevant human impacts.

This is the first baseline survey of the taxonomic and resistance potential of microbial communities in Puget Sound. Using high-throughput and sequence-based comparative metagenomics, we identified common taxonomic and antibiotic resistance determinant signatures for the open Puget Sound locations. Comparison of these metagenomic profiles between the open Sound, a nearshore marina and effluent from a proximal WWTP revealed unique profiles across the different environments that follow a gradient of human impact. This investigation provides an initial framework by which to monitor the marine environment for genomic determinants relevant to public health.

## Materials and Methods

### Ethics Statement

All necessary permits were obtained for the described field studies. This statement applies to the collection of the wastewater treatment plant (WWTP) effluent sample. Permission for the WWTP sample was granted by the West Point Treatment Plant (King County, WA), specifically Betsy Cooper (NPDES Administrator, Wastewater Treatment Division, King County Department of Natural Resources and Parks) and Rick Hammond (Chief Process Analyst, West Point Treatment Plant). No specific permits or permissions were required for the water samples collected in Puget Sound. These cruise sample locations are routinely monitored by the University of Washington Puget Sound Regional Synthesis Model (PRISM) program, and are not privately owned or protected in any way. The marina sample was taken from a public boat ramp run by the City of Seattle. These field studies did not involve endangered or protected species.

### Sample Collection

Surface water samples (∼5 m depth) were collected aboard the R/V Thomas Thompson from October 29–30, 2010 at five stations in Puget Sound, WA (Figure 1). These stations have been monitored since 1998 by the University of Washington Puget Sound Regional Synthesis Synthesis Model (PRISM) program. A nearshore surface water sample was also collected at Shilshole Bay Marina in Seattle, WA on December 20, 2010 and an effluent sample from the King County West Point Treatment Plant (WWTP) in Seattle, WA was collected on January 31, 2011. The Marina is located approximately 8 miles north of central Seattle and 2 miles north of the WWTP, and includes 1,400 moorage slips (300 slips for liveaboards), public water access and a public promenade. The West Point Treatment Plant has an average daily inflow of 133 million gallons, and the wastewater is sourced from stormwater/groundwater (53%), residential (29%), commercial (17%) and industrial (1%) processes [38]. At each station and sampling location, 80 liters of water were pumped through a peristaltic pump system (Cole-Palmer, U.S.A.) and filtered sequentially (i.e. size fractionated) through 3-µm (147 mm) polycarbonate membranes (Sterlitech, WA) and 0.2-µm (147 mm) Supor membranes (Pall Corporation, U.S.A). Filters were covered with sucrose lysis buffer (50 mMTris•HCl, 40 mM EDTA, and 0.75 M sucrose) and stored at −80°C on board and then transferred to −80°C in the laboratory. For the cruise samples, physicochemical conditions (e.g. temperature, salinity, oxygen, fluorescence) were measured using a conductivity-temperature-depth (CTD) sensor array mounted on a Niskin bottle rosette (Table 1).

**Table 1:** Sampling locations and summary statistics for the Puget Sound metagenomes.

Environmental data										
Sample	Location	Date	Depth(m)	Temp (°C)	Salinity (ppt)	Chlorophyll(mg/m^−3^)	No. ofreads	Mean readlength (bp)	% GC	No. of contigs (>1 kb)
P1	48°01′0′′N; 122°18′2′′W	10/29/10	5	11.5	30.02	0.2	187,776	367	43.8	81
P5	47°53′0′′N; 122°22′0′′W	10/29/10	5	11.53	30	0.51	409,672	372	43.2	1,277
P26	48°22′5′′N; 122°43′0′′W	10/29/10	5	9.5	30.6	0.3	146,173	367	43.3	6
P28	47°42′2′′N; 122°27′2′′W	10/29/10	5	11.51	32.2	0.15	164,069	369	44.2	22
P32	47°20′0′′N; 122°26′5′′W	10/30/10	5	11.48	30.15	0.58	159,314	367	43.6	12
Marina	47°41′2′′N; 122°24′25′′W	12/20/10	0.5	∼11.00^b^	∼24.00^b^	No data	228,712	379	46.8	86
WWTP	47°39′42′′N; 122°26′0′′W	1/31/11	NA^a^	14.9	<0.5	No data	120,857	381	56.4	77

a NA, not applicable.

b Based on observational data.

### DNA Extraction and Sequencing

DNA was extracted from the 0.2-µm filters as these filters contain the bacterial fraction of the marine community. The filters were thawed and cut into eighths. Total metagenomic DNA was extracted using the Powerwater DNA Isolation Kit (Mo Bio Laboratories, CA) following the manufacturer’s protocol with modifications. This kit is specifically designed for isolating DNA from filtered environmental water samples and includes inhibitor removal technology aimed at removing humic acid and other organic matter commonly found in environmental samples that can interfere with downstream analyses. The protocol modifications included an incubation at 65°C for 10 minutes following addition of Solution P1 and a lysozyme digestion (final concentration = 10 mg/ml) at 37°C for 1 hour and an RNase digestion at 37°C for 20 minutes immediately following the bead-beating step. DNA quantity and quality were estimated using the Quant-iTPicogreen dsDNA Assay Kit (Invitrogen, U.S.A.) and the Nanodrop-1000 Spectrophotometer (Nanodrop Technologies, DE). DNA concentrations ranged from 75 to 130 ng/µl per sample. Gel electrophoresis showed high molecular weight DNA fragments 3–12 kb in size. Approximately 3 µg of total DNA for each sample was used for a multiplexed pyrosequencing run on the Roche/454 GS FLX Titanium platform in the Department of Microbiology at the University of Washington.

**Figure 2:**
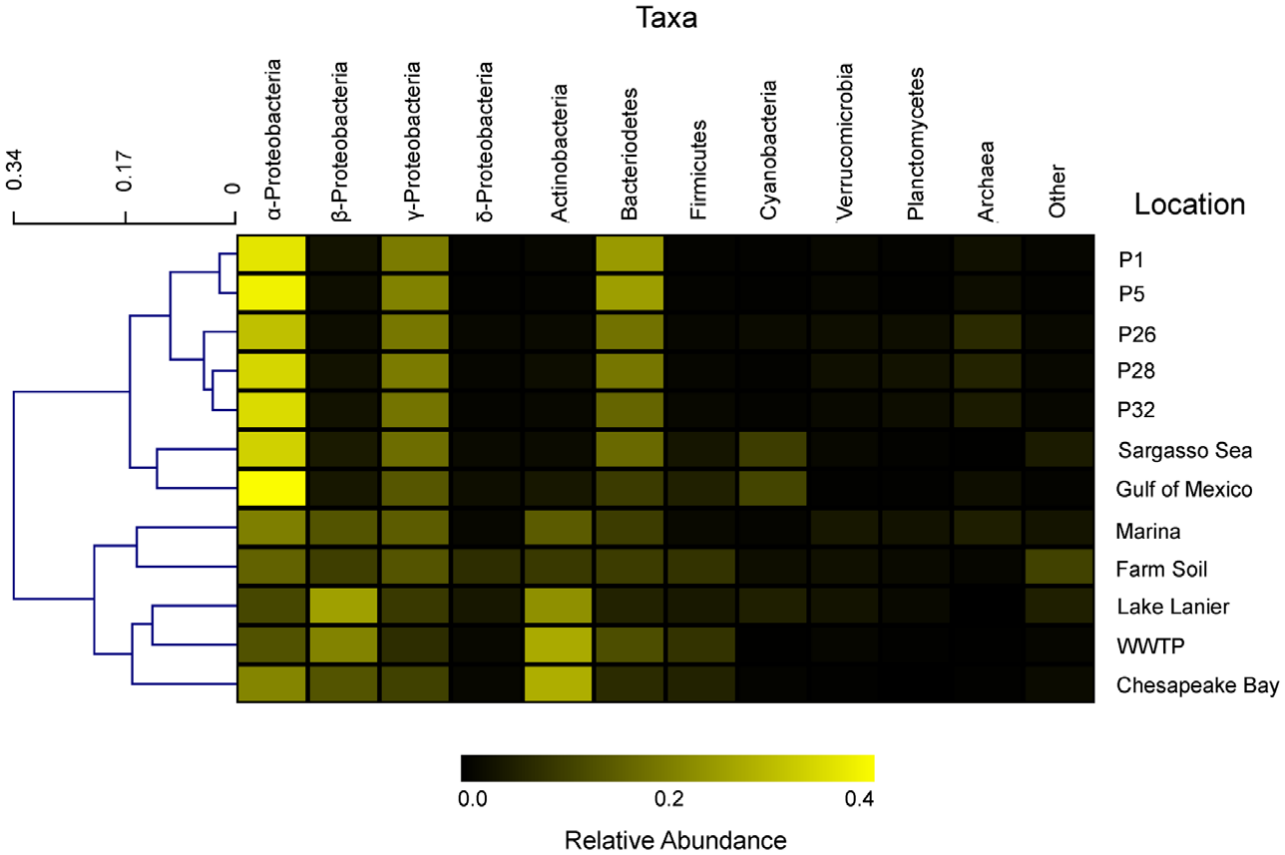
Relative abundance of major taxonomic groups in the Puget Sound samples and other selected metagenomes. Taxonomic groupings were based on BLASTX comparison to the NCBI taxonomy using MG-Rast [39] and ≥50% identity and an alignment length ≥50 amino acids. The ‘other’ category includes bacteria taxa present in <1% of sequences in all samples, eukaryotes and viruses. Shading is proportional to the relative abundance of each taxon within a metagenome. The cladogram was displayed using hierarchical clustering and the Euclidian distance metric. See Materials and Methods for references describing the additional metagenomes used for comparison. The open Sound locations cluster with other more saline environments including open and coastal ocean samples while the Marina and WWTP effluent samples cluster within a larger clade containing other freshwater and freshwater-impacted metagenomes.

**Figure 3:**
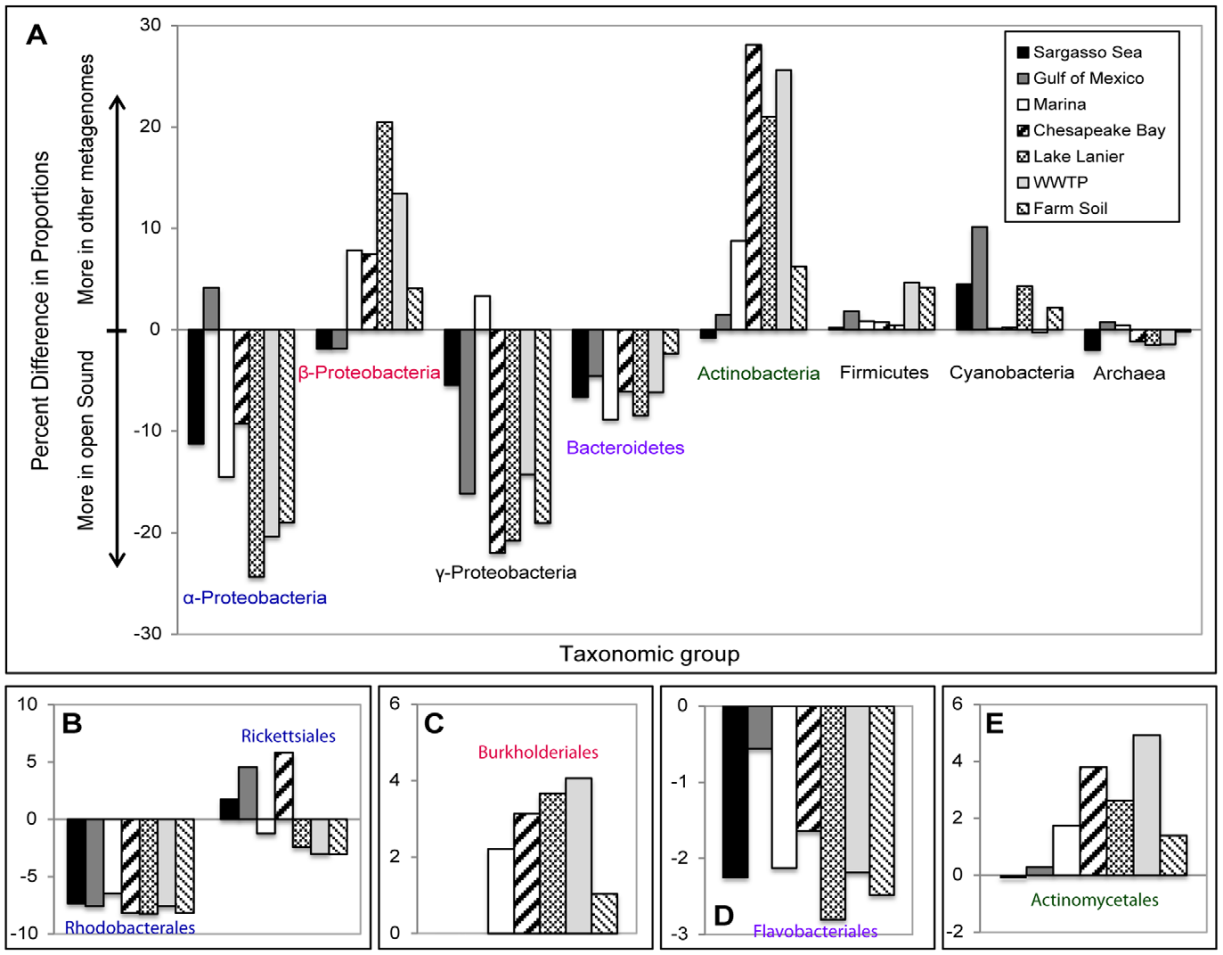
Over- and under-representation of predominant taxa in selected biomes relative to the open Puget Sound metagenome. (A) Overall taxa, (B) α-Proteobacteria, (C) β-Proteobacteria, (D) Bacteroidetes and (E) Actinobacteria. The difference in proportions refers to the percent difference in the relative abundances of a given taxa between two locations. Only taxa with a difference of proportions >1% are shown. Negative values indicate higher relative abundance in the Puget Sound (p<10^−15^ for all comparisons). See Materials and Methods for references describing the additional metagenomes used for comparison. A unique taxonomic signature was identified for the Puget Sound consisting of an over-representation of α-Proteobacteria, γ-Proteobacteria and Bacteriodetes and an under-representation of β-Proteobacteria and Actinobacteria.

### Bioinformatic Analyses

The 454 sequence reads were initially trimmed to remove barcoded sequences and any secondary adapter sequences. Reads containing <5% of any one nucleotide were also removed. For each sample, raw reads were assembled into contigs using Newbler v. 2.5.3 (Roche Diagnostics-454 Life Sciences) with default settings except for a more stringent 95% minimum overlap identity. For taxonomic annotation, the unassembled reads were run through the Meta Genome Rapid Annotation using Subsystems Technology (MG-RAST) pipeline [39]. This pipeline includes QC steps to eliminate low quality sequences. Reads meeting any of the following three criteria were omitted: read length >2 standard deviations from the mean sample read length, read containing >5 ambiguous bases or reads identical to another sequence over the first 50 bp. Unassembled reads were taxonomically annotated using the lowest common ancestor (LCA) algorithm and only sequence matches with ≥50% identity over ≥50 amino acids were retained. This algorithm assigns each read to the LCA within the set of matching taxa from the BLASTX search [40]. For example, if a given read had sequence similarity to 3 different families within the same order, the read is assigned at the order level. The LCA algorithm is thus predicted to have a lower rate of false positive assignments than the best hit BLAST approach but with a higher number of unspecific assignments or no hits. Genus level annotation was performed using 16S rRNA analysis. An *Escherichia coli* 16S rRNA reference sequence was searched against the 454 reads using BLASTN (default settings) and matching 454 reads were run through the Ribosomal Database Project (RDP) Classifier [41] to classify the 16S rRNA sequences. Only those classifications with at least a 50% confidence estimate were included in the analysis. Additional environmental metagenomes used for taxonomic comparison to the Puget Sound dataset included: Sargasso Sea (GS001a, GS001b and GS001c), Chesapeake Bay (GS012) and the Gulf of Mexico (GS016) from the Global Ocean Sampling Expedition [42], Lake Lanier [43] and farm soil [44]. All the water samples were collected in 1–5 m depth.

**Figure 4:**
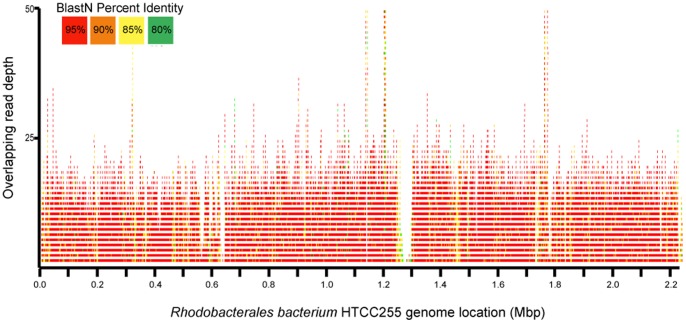
Recruitment plot of the unassembled Puget Sound reads to the alpha-Proteobacterium HTCC2255 genome assembly. Open Sound and Marina metagenomic sequence reads were recruited to the 2.23 MB assembly (GI number: 211594581) using BLASTN and an E-value cutoff of 10^−5^. Over 91% of the assembly was matched at greater than 95% identity, equaling approximately 7X coverage.

**Figure 5:**
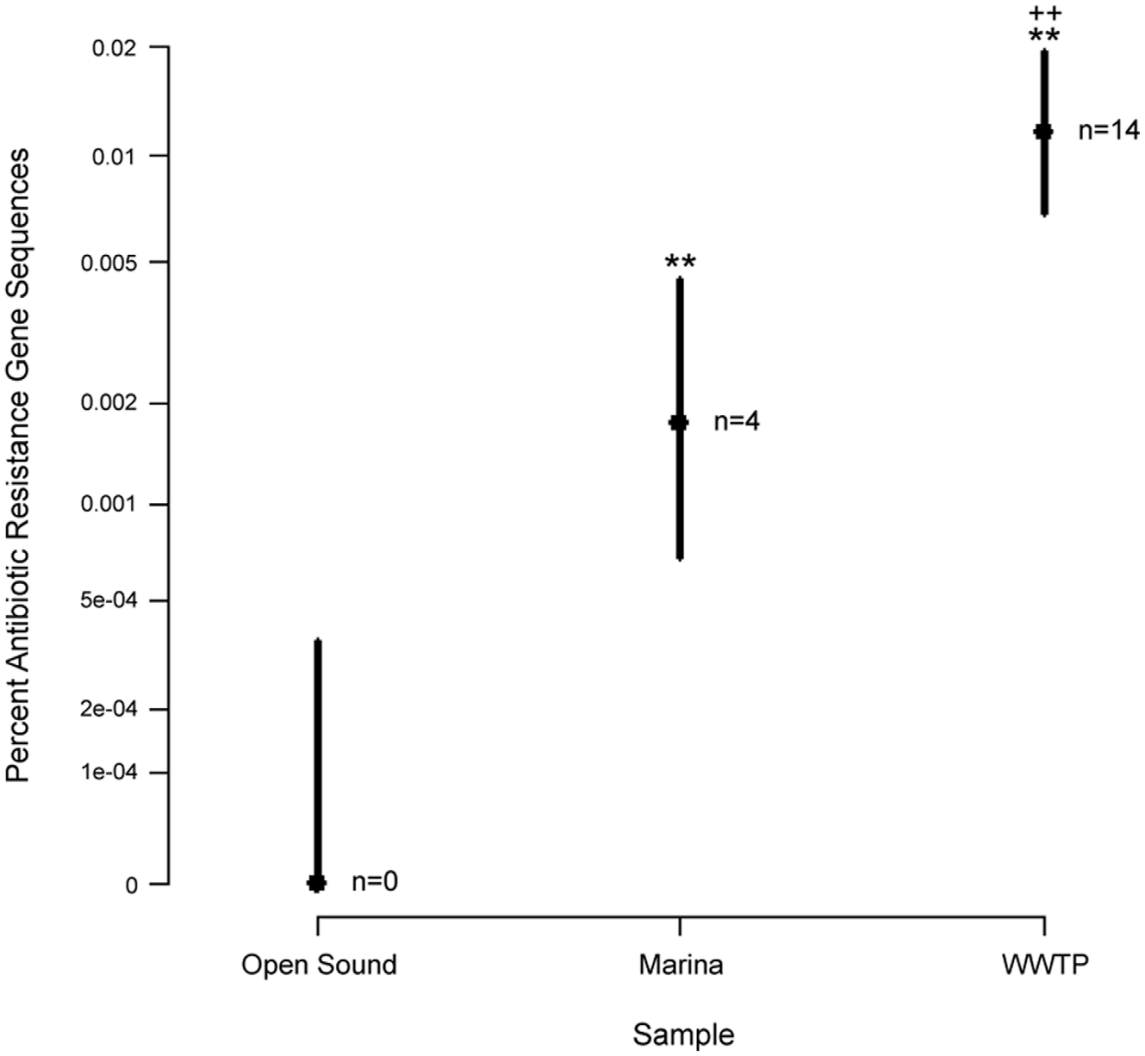
Prevalence of antibiotic resistance gene sequences in the Puget Sound metagenomes. Sequences were classified as antibiotic resistance genes if sharing ≥80% identity and an alignment length ≥50aa to a sequence within an expanded Antibiotic Resistance Genes Database (ARDB) [45]. Open Sound samples were pooled together for the analysis. Bars represent 95% confidence intervals for binomial proportions. For open Sound vs. Marina and WWTP, *p<0.05 and **p<0.005. For Marina vs. WWTP, ^+^p<0.05 and ^++^p<0.005. ‘n = ’ refers to the number of antibiotic resistance gene sequences. The y-axis represents a modified log scale. While the resistance gene signals were low for the samples, the signals were significantly different across the sample types, suggesting antibiotic resistance gene abundance may reflect differences in potential human health impacts.

Read recruitment to alpha proteobacterium HTCC2255 was performed using BLASTN (E-value <10^−5^). The 2.23 Mbp genomic scaffold (GI number: 211594581) was downloaded from GenBank. The recruitment plot was generated using in-house custom visualization software.

**Figure 6:**
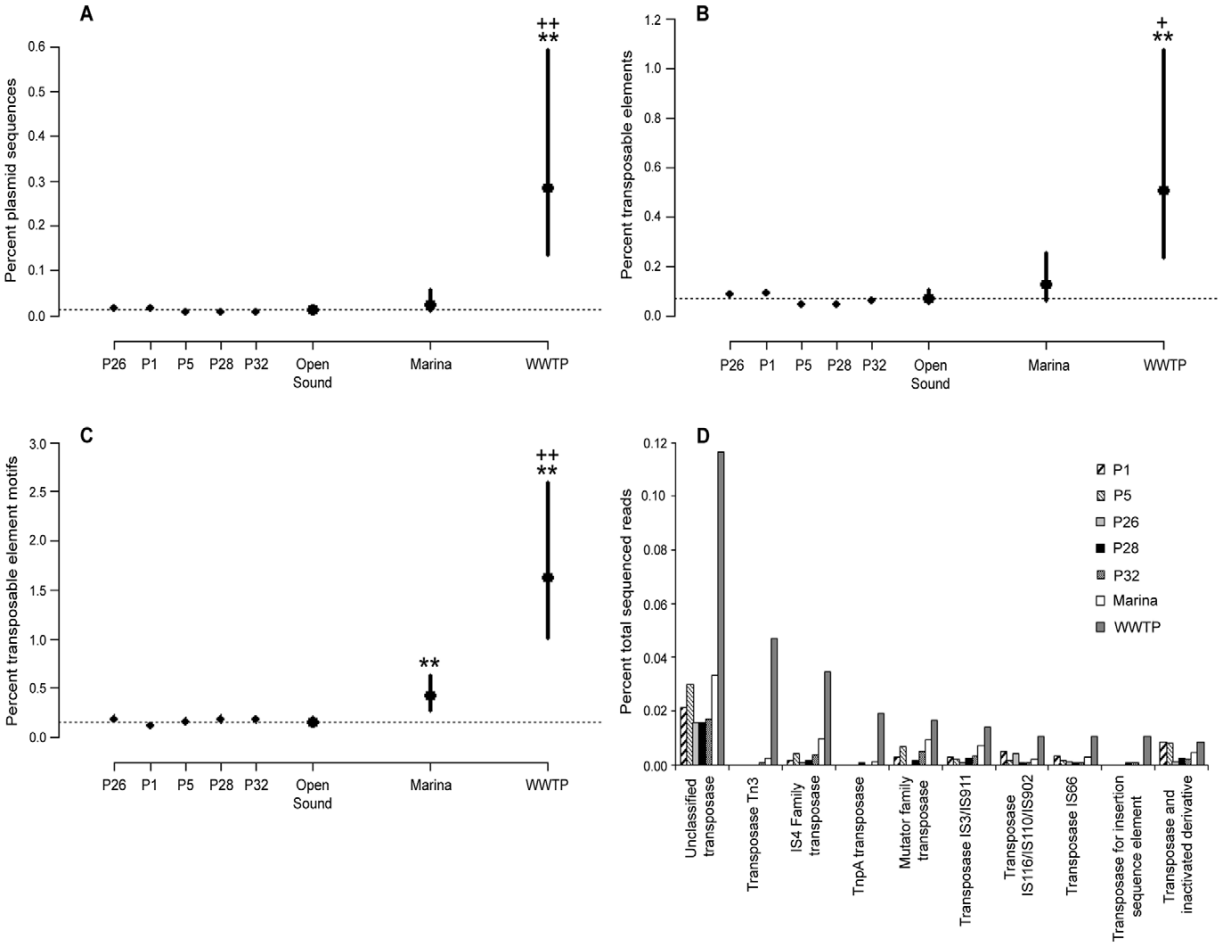
Prevalence of mobile genetic elements in the Puget Sound metagenomes. (A) Plasmid sequences, (B) transposable elements using a BLAST search against GenBank, (C) transposable elements using a hidden markov model (HMM) search against the Pfam database and (D) top ten most abundant transposable elements from the WWTP effluent by percent abundance. Bars represent 95% confidence intervals for binomial proportions. P-values are relative to the open Sound and are corrected for multiple testing. For open Sound vs. Marina and WWTP, *p<0.05 and **p<0.005. For Marina vs. WWTP, ^+^p<0.05 and ^++^p<0.005. Bars represent 95% confidence intervals for binomial proportions. The dashed line represents the mean percent for the open Sound. Similar profiles for mobile genetic elements were seen for the open Sound samples while significant differences emerged when comparing to the Marina and effluent.

To identify putative antibiotic resistance genes, an expanded version of the Antibiotic Resistance Gene Database (ARDB) [45] was generated that incorporates all sequences deposited in GenBank to date that are ≥80% identical to sequences already in the ARDB. The ARDB is a commonly used database consisting of over 23,000 resistance genes from nearly 1,700 species. A nonredundant version of the ARDB was first compiled and consisted of 3,185 sequences. An expanded dataset, termed the ARDB+, was generated by searching the sequences from this nonredundant list against GenBank using the 80% identity cutoff [45]. The ARDB+ increases the number of nonredundant ARDB sequences to a total of 11,500 sequences. A list of 103 antibiotic resistance gene sequences from metagenomic samples that were functionally verified to confer resistance [28], [29], [30], [33] was also compiled and included in the ARDB+. MetaGeneMark [46] (default settings) was used to predict potential protein coding sequences for the unassembled Puget Sound and WWTP reads, and these predicted peptides were then BLASTP searched [47] against the ARDB+ (E-value <10^−5^). Reads with ≥80% identity over an alignment length ≥50 amino acids to a resistance gene within the ARDB+ were annotated according to the best hit.

Plasmid sequences were annotated by aligning the unassembled reads and contigs against the plasmid sequences available in the NCBI RefSeq database (1843 sequences) using BLASTN (E-value <10^−5^). Only those sequences with a nucleotide sequence identity ≥95% over an alignment length of at least 100 bp were retained [32], [48].

Two approaches were used to identify transposable elements in the dataset. First, 167 unique genes annotated as transposable elements were downloaded from GenBank. Unassembled reads were then compared against these sequences using BLAST, and reads with a sequence identity ≥80% [32] over an alignment length of at least 150 bp were annotated as transposable elements. Second, unassembled reads were compared to 41 protein motifs annotated as transposable elements within the Pfam 26.0 database [49] using an E-value cutoff of 10^−10^. Pfam is a database of conserved protein families and domains across species, where the domains represent structural and functional motifs of the protein. A significance threshold of E<10^−10^ represents a more stringent cutoff than the manually curated Pfam gathering threshold or trusted cutoff, and thus minimizes the number of false positive assignments. The abundance of transposable elements was normalized to total sequence reads per sample for the BLAST approach and to total motif counts per sample for the Pfam approach.

### Statistical Analyses

Overrepresentation or underrepresentation of taxonomic units (domain, phylum or class) was determined using the Statistical Analysis of Metagenomic Profiles (STAMP) software package [50]. Only taxonomic units that had an effect size >1 between two metagenomes were included in the statistical analysis. P-values were calculated using the two-sided Fisher’s Exact test, and 95% confidence intervals were calculated with the Newcombe-Wilson method. The Benjamini-Hochberg FDR method [51] was used to correct for the false discovery rate. Hierarchical clustering of metagenomes by taxonomic units using the Euclidian distance metric was performed with the TIGR Multiexperiment Viewer (TMeV) (http://www.tm4.org/mev/). Counts were normalized to total taxonomic counts to give a measure of relative abundance within a metagenome. Significance tests were run to determine whether there were statistical differences in the abundance counts across samples. Data was analyzed using a generalized linear model for binomially distributed data. Differences among open Sound samples were treated as a source of extra-binomial variability in the statistical model to provide estimates of variability for the open Sound, Marina and WWTP samples in addition to the simple binomial variability based on the counts [52].

### Metagenome Sequence Accession

Sequence data for the Puget Sound dataset is available through the Metagenomics RAST (MG-RAST) server (http://metagenomics.anl.gov/) under the MG-RAST identification numbers 4460178.3, 4460179.3, 4460180.3, 4460182.3, 4460188.3, 4460189.3 and 4460190.3.

## Results and Discussion

Shotgun metagenomics in combination with pyrosequencing were used to explore the taxonomic and antibiotic resistance determinant composition at six locations from north to south Puget Sound in addition to an effluent sample from a WWTP that deposits into the main basin of Puget Sound. The environmental data and summary statistics for the pyrosequencing run are shown in Table 1. Approximately 1.4 million reads were generated that passed the QC filter, comprising nearly 530 million base pairs of sequence data. There were 22 Mbp of assembled contigs greater than 500 bp and 47 contigs greater than 10,000 bp. The N50 contig size was 1.1 kb.

### A Taxonomic Signature of Puget Sound

Approximately 42% of the unassembled sequence reads were assigned by phylum to protein sequences within GenBank using our sequence similarity criteria of ≥50% identity and an alignment length ≥50 aa. Bacteria accounted for 93% of all annotated sequence reads, with Archaea (4%), Eukaryota (2%) and viruses (1%) comprising the remaining proportion. All communities were dominated by the phylum Proteobacteria, which accounted for 49–67% of all sequence reads (Figure 2). As shown by the positive and negative bars in Figure 3, the open Sound had an overrepresentation of α- and γ-Proteobacteria and Bacteriodetes and a decreased proportion of β-Proteobacteria, Actinobacteria and Firmicutes when compared to the Marina, WWTP and other metagenomes (p-value <10^−15^ for all comparisons). The Marina exhibited a similar pattern of taxon overrepresentation when compared to the WWTP except on a smaller magnitude. The dominance of α-Proteobacteria and γ-Proteobacteria and low abundance of β-Proteobacteria in the open Sound samples has also been characterized in other open and coastal ocean locations [53], [54], but α- and γ-Proteobacteria appear to represent an even larger proportion of total taxa in the open Sound samples than the other marine metagenomes included in this study except for α-Proteobacteria in the Gulf of Mexico.

The overrepresentation of α-Proteobacteria in the open Sound was due to a high abundance of *Rhodobacterales sp.* (Figure 3; Figure S1). A total of 47,425 sequence reads from the open Sound and Marina were recruited to 92% (7x coverage) of the *Rhodobacterales bacterium* HTCC2255 reference genome assembly with a minimum 95% identity per read (Figure 4). This genome was also previously assembled from a metagenomic sample taken from Puget Sound [55]. The high abundance of *Rhodobacterales* is in contrast to the other marine metagenomes where *Rickettsiales sp.*, another group of α-Proteobacteria that includes the ubiquitous SAR11 clade, was the dominant organism (Figure S1). Recent metatranscriptomic samples collected from Monterey Bay, CA also contained a high abundance of *Rhodobacterales sp.* HTCC2255 and a large *Rhodobacterales*: *Rickettsiales* ratio [56], suggesting *Rhodobacterales* may be a dominant organism in surface waters on the U.S. West coast. Members of the phylum Bacteroidetes have also been shown to be widespread in marine environments, and the increased proportion of this taxon in the open Sound relative to the other metagenomes is attributable to an overrepresentation of *Flavobacteriales sp.* (Figure 3). The proportion of Actinobacteria significantly increased from ∼2% of reads in the open Sound to 13% in the Marina and 25% in the WWTP sample (p-value <10^−15^ for all comparisons). Actinobacteria was also overrepresented in the other freshwater and freshwater-impacted environments, and further analysis revealed a strong negative correlation between Actinobacteria abundance and salinity (r = **−**0.952; p<10^−6^) (Figure S2). Actinobacteria in coastal areas has previously been suggested to be a potential signal of terrestrial or freshwater runoff [11], [57], and our results support this notion. α-Proteobacteria (r = 0.904; p<10^−4^), β-Proteobacteria (r = −0.913; p<10^−5^), γ-Proteobacteria (r = 0.860; p<10^−4^), Bacteroidetes (r = 0.612; p<0.05) and Firmicutes (r = −0.644; p<0.05) were also correlated with salinity, while cyanobacteria abundance was correlated with temperature (r = 0.804; p<0.005). The GC-content distribution also reveals the taxonomic similarity between the open Sound samples and the differences in community composition between the open Sound, Marina and WWTP effluent (Table 1). The open Sound had a mean %GC of 43.62%±0.40, with a slight increase for the Marina sample (46.8%). The GC-content of the WWTP sample was significantly higher than for all other samples (56.4%).

Approximately 3.3% of sequence reads were classified at the genus level by the RDP. Open Sound samples were characterized by a high abundance of *Candidatus Pelagibacter* (SAR11 clade) and *Thalassobacter* (family Rhodobacteraceae) from the phylum α-Proteobacteria and *Polaribacter* from Bacteroidetes. The Marina was dominated by the γ-proteobacterium *Glaciecola*, accounting for 28.3% of genera diversity. *Glaciecola* was absent in all other samples except P5 where it comprised 0.7% of genera diversity. The abundance of bacteria from the genus Acinetobacter, which has been linked to hydrocarbon degradation in marine environments and has been shown to be seasonally prevalent in a coastal marina [11], was not significantly different between the Marina (0.1%) and open Sound (∼0.03–0.07%) but was considerably elevated in the WWTP (1.07%). The WWTP effluent also contained more specific taxonomic markers of potential human impact. Firmicutes, which is one of the dominant phyla of the human gut [58], accounted for a significantly greater proportion (8.4%) of all WWTP phyla than in the other samples except farm soil, and was also associated with a number of the WWTP putative antibiotic resistance gene sequences. *Clostridiales sp.* was the most abundant group within Firmicutes, members of which are commonly found in human or animal feces [59], [60]. *Blautia* is a core human gut microbe [61], and *Hespellia* is a member of the family *Lachnospiraceae* within which phylotypes have been identified as genetic markers of human fecal contamination [62]. Other taxonomic surveys of WWTP environments have also found an increased abundance of Firmicutes [63], [64], and one recent study used the ratio of Firmicutes and Bacteroidetes to α-Proteobacteria abundance as an indicator of fecal pollution in watersheds [12]. Thus the overabundance of Firmicutes within the WWTP distinguishes this environment from the other marine and freshwater metagenomes, and may suggest the use of this phylum as a potential indicator of human impact.

Despite the spatial variation between the open Sound samples, the taxonomic composition across these surface water samples was strikingly similar and can likely be attributed to similar salinity and temperature gradients. Salinity has been shown to be one of, if not the most, important environmental parameters determining the level of similarity between isolated microbial communities [65]. This is evidenced by the fact that the samples cluster by salinity (Figure 2). The open Sound samples cluster with other more saline environments including the Sargasso Sea (open ocean) and the Gulf of Mexico (coastal ocean), while the Marina and WWTP effluent cluster within a larger clade that contains other freshwater and freshwater-impacted metagenomes (Lake Lanier, Chesapeake Bay and farm soil). The freshwater clade can be described by a high proportion of Actinobacteria, an increased abundance of β-Proteobacteria and an overall increased level of diversity. The metagenomes composing this freshwater clade can also be defined as being human impacted environments, and while it is not possible to further tease apart the potential influence freshwater input versus human impact has on the taxonomic profiles, it is important to note that the two are related in that environmental pollutants can enter marine ecosystems via freshwater runoff and thus a freshwater signal may in some cases be a potential indicator of human impact.

It is also important to note the potential impact temporal differences may have had on community composition. Seasonality has been shown to be a strong driver of marine microbial composition [66]. While the open Sound samples were collected within a span of two days at the end of October, the Marina and effluent samples were collected approximately 7 and 12 weeks later respectively. By nature of the fact that the freshwater effluent sample is already taxonomically distinct from the marine samples due to salinity and source, temporal variability is not likely a primary explanatory variable for describing the differences in community composition. Seasonal influences are more relevant when comparing the open Sound to the Marina. Freshwater discharges from rivers into Puget Sound are at a maximum in late autumn due to rainfall runoff and this flow continues at elevated levels through the winter [35]. The lower salinity of the December Marina sample (∼24 ppt) may therefore correspond to increased freshwater inputs. Subsequently the community composition of the Marina sample, which includes a higher proportion of the potentially freshwater or terrestrial-sourced Actinobacteria, may reflect these seasonal differences. The temperatures of the Marina and open Sound samples were similar despite the two month lag (Table 1). More temporal monitoring data is needed to better explain the role seasonality has on bacterial composition at these locations.

### Proportion of Putative Antibiotic Resistance Gene Sequences Differs Across Metagenomes

In order to determine the abundance of putative antibiotic resistance genes in the Puget Sound and WWTP datasets, the sequence data was searched against an expanded and nonredundant ARDB (termed ARDB+) which also included 103 functional antibiotic resistance genes sequences isolated from metagenomic projects. Approximately 0.0013% (n = 18) of all sequence reads had ≥80% identity and an alignment length ≥50 aa to a sequence within the ARDB+. To validate this annotation, the 18 sequences were also searched against GenBank, and results indicated that the best protein hits within GenBank were also to antibiotic resistance genes. Of these 18 sequences, the WWTP effluent had the highest representation (n = 14), followed by the Marina (n = 4) and the open Sound (n = 0) (Figure 5; Table S1). For comparison, the proportion of putative resistance gene sequences in the WWTP effluent (0.012%) was lower than that for other pyrosequenced metagenomes from different environmental matrices including a river sediment sample taken downstream from an Indian WWTP that processes large quantities of drugs (1.4%) [32] and the plasmid fraction of a WWTP activated sludge sample (1.1%) [31], but slightly greater than that of an activated sludge metagenome from another municipal WWTP (0.008%) [15]. Tetracycline resistance genes had the highest representation (33%) within the 18 putative antibiotic resistance gene sequences found in our samples. The WWTP putative antibiotic resistance gene sequences had similarity to those from clinically relevant bacteria species including *Clostridium*, *Enterococcus* and *Streptococcus*, while resistance gene sequences from the Marina were similar to those from environmental bacteria including *Desulfococcus*, *Polaromonas* and *Pseudomonas*. The average percent protein coverage based on the sequence alignment of the 454 reads to ARDB+ sequences was 33.8%±18.9 (mean±SD).

There was a significant increase in the proportion of putative resistance gene sequences from the open Sound to Marina to WWTP (Figure 5), suggestive of a relationship between resistance gene abundance and human impact across these environments. The fact that a differential resistance gene signal could be detected across the different environments supports using a pyrosequencing approach to identify trends in resistance gene abundance across diverse environments. Challenges still remain though in using this approach for quantification of resistance genes in metagenomes. First, the low sequencing depth per sample likely limits our ability to detect the full resistance gene repertoire of complex microbial community samples. Depth of sequencing is a significant limitation in metagenomic investigations and thus targeting specific genomic elements in whole-genome sequence data has been a considerable challenge [67]. This is especially relevant for aquatic samples where dilution reduces the likelihood of identifying specific genes. That only 18 sequences in the Puget Sound dataset matched to known resistance genes potentially suggests a considerable under-sampling of the resistome when using pyrosequencing. Other marine and freshwater pyrosequenced metagenomes also have a relatively rare representation of resistance genes (Figure S3), suggesting the need for higher sequencing depths than achievable by 454 technology to fully characterize the resistomes of surface water microbial communities. For example, Illumina sequencing technology allows for greater sequencing depth than does pyrosequencing, but with shorter read lengths and therefore a greater reliance on sequence assembly, which still remains a challenge for mixed microbial communities. Secondly, the lack of sequence data for the unculturable prokaryotic majority in combination with evidence from functional metagenomic studies indicating that many environmental resistance genes appear to be distantly related and share low sequence similarity to cultured resistance genes may result in an underestimation of the actual number of resistance genes. This challenge is being addressed through further sequencing of bacterial reference genomes and functional metagenomic projects, however more research is needed. Lastly, sequence-based identification of resistance genes does not necessarily imply these genes are functionally expressed. The resistance potential can be estimated using sequence-based metagenomics, but functional metagenomics or transcriptional analysis is needed to detect functional resistance genes and more clearly define public health risks.

The fate of antibiotic resistance determinants during the wastewater treatment process is relatively unknown at this time. As no influent from the WWTP was collected in this study, it is unclear what impact the wastewater treatment process had on the prevalence of antibiotic resistance genes in the effluent. Previous studies have shown mixed results regarding resistance gene levels in pre- versus post-treated sewage. While concentrations of resistance genes in wastewater may significantly decrease through the wastewater treatment process [68], other studies have found that resistance gene levels are higher in the effluent and that therefore the treatment process may be selecting for resistant bacteria, genes or mobile genetic elements [69], [70], [71]. Either way, the effluent had a significantly higher abundance of antibiotic resistance genes than Puget Sound, although further effluent sampling is needed to support this individual finding.

### Differential Abundance of Mobile Genetic Elements Across Metagenomes

Mobile genetic elements including plasmids and transposable elements are important vectors for the transfer of antibiotic resistance genes [72], [73]. Although in-depth profiling of the plasmid fraction of metagenomes is currently not possible for whole genome sequencing projects due to low depth of sequence coverage, insights into the plasmid composition of the Puget Sound and WWTP metagenomic datasets could still be made from the ∼0.04% of sequences that matched known plasmids. A total of 124 sequence reads (0.01%) and 3 contigs from the open Sound and Marina metagenomes and 265 reads (0.22%) and 4 contigs from the WWTP matched plasmids within the NCBI RefSeq database using a similarity criteria of ≥95% sequence identity over 100 bp (Figure 6A; Table S2 and Table S3). There were 13 plasmids in common between the open Sound, Marina and WWTP metagenomes. The open Sound and Marina plasmid sequences were dominated by α- and γ-Proteobacteria, while the WWTP plasmids were associated with α-, β- and γ-Proteobacteria, Firmicutes and Actinobacteria. At the species level, nearly half of the plasmid matches to the open Sound and Marina reads were sourced from *Silicibacter sp. Silicibacter* is a member of the marine Roseobacter clade (α-Proteobacteria) and has been shown to form symbioses with phytoplankton [74]. The remainder of the open Sound and Marina plasmid sequences had taxonomic assignments to other environmental bacteria in addition to a number of human pathogens including *Klebsiella pneumonia*, *Salmonella enterica*, *Vibrio sp.*, *Pseudomonas aeruginosa* and *Serratia marcescens*. Many of these plasmids contain genes associated with virulence factors, heavy metal resistance, beta-lactamase, tetracycline and aminoglycoside resistance and antibiotic resistance determinants including transposases and integrons. For example, plasmid TC68 from *Vibrio sp.* is a known chloramphenicol resistance gene that has been previously isolated from a fish farm microbial community [75]. The WWTP plasmid sequences were characterized by both pathogenic and non-pathogenic host-specific bacteria. Human pathogens included *Bacteroides fragilis, Campylobacter*, *Enterococcus*, *Klebsiella pneumonia*, *Listeria monocytogenes* and *Salmonella*. Also present were bacterial indicators of fecal contamination, including *Bifidobacterium* and *Escherichia coli*. Bacteria associated with food products and used in food production were also prevalent, including *Lactococcus lactis*, *Lactobacillus rhamnosus* and *Streptococcus thermophiles*. The two largest contigs from the WWTP that matched plasmid sequences had 100% identity to plasmids from uncultured bacteria previously identified in activated sludge from other WWTPs [76], [77] (Table S2).

Both approaches to identify transposable elements resulted in an increasing abundance of transposable elements from open Sound to Marina to WWTP, with a highly elevated proportion in the WWTP (Figure 6B and 6C). The Pfam approach did detect a higher proportion of transposable elements in the Marina and WWTP than the BLAST approach, as well as a significant difference in transposable element abundance between the Marina and open Sound. No significant differences in the abundance of transposable elements were seen within the open Sound samples using either approach. The most abundant transposases in the effluent included Tn3, IS4, TnpA (gene product of IS608), the mutator transposases and IS3 (specifically IS911), in addition to a large percentage of unclassified transposases (Figure 6D). While the most abundant transposable elements in the Puget Sound samples were the same as that for the effluent, they were for the most part present in much lower numbers. Transposable elements from the effluent were more likely to be found on plasmids than the Puget Sound samples. Approximately 20.7% of transposable elements from the effluent were annotated as being sourced from plasmids, compared to 0.039%±0.008 for the open Sound and 13.56% for the Marina. Plasmids that were unique to and represented at least 3% of the total plasmids annotated as carrying transposable elements in the WWTP sample included pA81 (biodegradation/heavy metal resistance) [78], pMOL28 (heavy metal resistance) [79] and pSKYE1 (aromatic compound degradation) [77]. Plasmid pAA1 (xenobiotic compound metabolism) [80] was also abundant in both the WWTP and Marina samples. Plasmids pDSHI01 from *Dinoroseobacter* and pMAQU02 from *Marinobacter* were commonly found within the Puget Sound samples.

This comparative metagenomic survey of Puget Sound has provided baseline data describing the community composition and antibiotic resistance determinant abundance across this marine environment in addition to an effluent sample from a proximal WWTP. Our results support the use of whole-genome pyrosequencing for comparing community composition across differentially impacted environments and for profiling antibiotic resistance determinants in highly impacted environments. To more completely capture the resistomes of natural environments with low human impact, sequencing technologies allowing for greater depth of sequencing may be needed. This study has highlighted the similarity of these metagenomic components in the open estuarine environment and the differences that emerge when analyzing more nearshore and human impacted ecosystems. Taken together, these results warrant further investigation into the potential for WWTP effluent to disseminate resistance determinants into the marine environment. As only one Marina and one WWTP sample were included in this study, further sampling of these environments is needed in order to support the observed trends. Furthermore, this study only analyzed surface water communities, and community composition has been show to change significantly with depth [81]. Increasing the spatial and temporal extents of metagenomic sampling and analysis will thus be important for longitudinal monitoring and further assessment of human impacts in marine environments.

## Supporting Information

Figure S1 **Relative abundance (order level) of major taxonomic groups in the Puget Sound samples and other selected metagenomes.** Sequences were assigned to the NCBI taxonomy using MG-Rast [39] and the lowest common ancestor algorithm (≥50% identity and alignment length ≥50 amino acids). Taxa representing >1% of assignable sequences in one or more samples are shown, while taxa present in <1% of sequences in all samples are grouped in the ‘other’ category.
(TIF)

Figure S2 **Relationship between the relative abundance of predominant taxa and salinity and temperature gradients.** Linear regression lines and Pearson’s coefficients (*p<0.05 and **p<0.005) are shown. Data points include the open Sound, Marina, WWTP, Chesapeake Bay, Sargasso Sea, Gulf of Mexico and Lake Lanier metagenomic samples.
(TIF)

Figure S3 **Abundance of antibiotic resistance gene sequences in environmental metagenomes with varying sequencing depth.** These metagenomes represent a diverse mix of environments, including river sediment samples taken downstream from a wastewater treatment plant (WWTP) processing high volumes of antibiotics [32], coastal surface water samples taken as part of the Global Ocean Sampling Expedition [82], the activated sludge fraction of a WWTP [15] and an urban freshwater lake [43]. Reads that aligned to a sequence within the ARDB+ with ≥80% sequence identity over at least 50 amino acids were classified as putative resistance genes.
(TIF)

Table S1 **Puget Sound 454 sequence reads with ≥80% similarity and an alignment length ≥50 amino acids to sequences within the expanded Antibiotic Resistance Genes Database (ARDB+).**
(PDF)

Table S2 **Plasmid sequences in the NCBI RefSeq database that match contigs from the Puget Sound and wastewater treatment plant (WWTP) effluent metagenomic datasets.**
(PDF)

Table S3 **Plasmid sequences in the NCBI RefSeq database that match sequence reads from the Puget Sound and wastewater treatment plant (WWTP) effluent datasets.**
(PDF)

## References

[1] United Nations Environment Programme (2006) Marine and coastal ecosystems and human well-being: a synthesis report based on the findings of the Millennium Ecosystem Assessment. UNEP. 76 p.

[2] Crain CM, Halpern BS, Beck MW, Kappel CV (2009) Understanding and managing human threats to the coastal marine environment. Ann N Y Acad Sci 1162: 39–62. 19432644 10.1111/j.1749-6632.2009.04496.x

[3] Halpern BS, Walbridge S, Selkoe KA, Kappel CV, Micheli F, et al (2008) A global map of human impact on marine ecosystems. Science 319: 948–952. 18276889 10.1126/science.1149345

[4] Kite-Powell HL, Fleming LE, Backer LC, Faustman EM, Hoagland P, et al (2008) Linking the oceans to public health: current efforts and future directions. Environ Health 7 Suppl 2S6. 10.1186/1476-069X-7-S2-S6 PMC2586713 19025677

[5] Laws EA, Fleming LE, Stegeman JJ (2008) Centers for Oceans and Human Health: contributions to an emerging discipline. Environ Health 7 (Suppl 2)S1. 10.1186/1476-069X-7-S2-S1 PMC2586714 19025672

[6] National Research Council (1999) From monsoons to microbes: Understanding the oceans role in human health. Washington, D.C.: National Academy Press. 166 p. 25101424

[7] Fleming LE, Broad K, Clement A, Dewailly E, Elmir S, et al (2006) Oceans and human health: Emerging public health risks in the marine environment. Mar Pollut Bull 53: 545–560. 16996542 10.1016/j.marpolbul.2006.08.012 PMC2573863

[8] Dinsdale EA, Edwards RA, Hall D, Angly F, Breitbart M, et al (2008) Functional metagenomic profiling of nine biomes. Nature 452: 629–632. 18337718 10.1038/nature06810

[9] Nogales B, Lanfranconi MP, Pina-Villalonga JM, Bosch R (2011) Anthropogenic perturbations in marine microbial communities. FEMS Microbiol Rev 35: 275–298. 20738403 10.1111/j.1574-6976.2010.00248.x

[10] Nogales B, Aguilo-Ferretjans MM, Martin-Cardona C, Lalucat J, Bosch R (2007) Bacterial diversity, composition and dynamics in and around recreational coastal areas. Environ Microbiol 9: 1913–1929. 17635539 10.1111/j.1462-2920.2007.01308.x

[11] Aguilo-Ferretjans MM, Bosch R, Martin-Cardona C, Lalucat J, Nogales B (2008) Phylogenetic analysis of the composition of bacterial communities in human-exploited coastal environments from Mallorca Island (Spain). Syst Appl Microbiol 31: 231–240. 18572341 10.1016/j.syapm.2008.04.003

[12] Wu CH, Sercu B, Van de Werfhorst LC, Wong J, DeSantis TZ, et al (2010) Characterization of coastal urban watershed bacterial communities leads to alternative community-based indicators. PLoS One 5: e11285. 20585654 10.1371/journal.pone.0011285 PMC2890573

[13] Tringe SG, Zhang T, Liu X, Yu Y, Lee WH, et al (2008) The airborne metagenome in an indoor urban environment. PLoS One 3: e1862. 18382653 10.1371/journal.pone.0001862 PMC2270337

[14] Hemme CL, Deng Y, Gentry TJ, Fields MW, Wu L, et al (2010) Metagenomic insights into evolution of a heavy metal-contaminated groundwater microbial community. ISME J 4: 660–672. 20182523 10.1038/ismej.2009.154

[15] Sanapareddy N, Hamp TJ, Gonzalez LC, Hilger HA, Fodor AA, et al (2009) Molecular diversity of a North Carolina wastewater treatment plant as revealed by pyrosequencing. Appl Environ Microbiol 75: 1688–1696. 19114525 10.1128/AEM.01210-08 PMC2655459

[16] Baquero F, Martinez JL, Canton R (2008) Antibiotics and antibiotic resistance in water environments. Curr Opin Biotechnol 19: 260–265. 18534838 10.1016/j.copbio.2008.05.006

[17] Taylor NG, Verner-Jeffreys DW, Baker-Austin C (2011) Aquatic systems: maintaining, mixing and mobilising antimicrobial resistance? Trends Ecol Evol 26: 278–284. 21458879 10.1016/j.tree.2011.03.004

[18] Martinez JL (2009) The role of natural environments in the evolution of resistance traits in pathogenic bacteria. Proc Biol Sci 276: 2521–2530. 19364732 10.1098/rspb.2009.0320 PMC2684669

[19] Aminov RI, Mackie RI (2007) Evolution and ecology of antibiotic resistance genes. FEMS Microbiol Lett 271: 147–161. 17490428 10.1111/j.1574-6968.2007.00757.x

[20] Salyers AA, Amabile-Cuevas CF (1997) Why are antibiotic resistance genes so resistant to elimination? Antimicrob Agents Chemother 41: 2321–2325. 9371327 10.1128/aac.41.11.2321 PMC164122

[21] Salyers AA, Gupta A, Wang Y (2004) Human intestinal bacteria as reservoirs for antibiotic resistance genes. Trends Microbiol 12: 412–416. 15337162 10.1016/j.tim.2004.07.004

[22] Allen HK, Donato J, Wang HH, Cloud-Hansen KA, Davies J, et al (2010) Call of the wild: antibiotic resistance genes in natural environments. Nat Rev Microbiol 8: 251–259. 20190823 10.1038/nrmicro2312

[23] McDaniel LD, Young E, Delaney J, Ruhnau F, Ritchie KB, et al (2010) High frequency of horizontal gene transfer in the oceans. Science 330: 50. 20929803 10.1126/science.1192243

[24] Soge OO, Meschke JS, No DB, Roberts MC (2009) Characterization of methicillin-resistant Staphylococcus aureus and methicillin-resistant coagulase-negative Staphylococcus spp. isolated from US West Coast public marine beaches. J Antimicrob Chemother 64: 1148–1155. 19837712 10.1093/jac/dkp368 PMC2782242

[25] D’Costa VM, King CE, Kalan L, Morar M, Sung WW, et al (2011) Antibiotic resistance is ancient. Nature 477: 457–461. 21881561 10.1038/nature10388

[26] Storteboom H, Arabi M, Davis JG, Crimi B, Pruden A (2010) Identification of antibiotic-resistance-gene molecular signatures suitable as tracers of pristine river, urban, and agricultural sources. Environ Sci Technol 44: 1947–1953. 20158229 10.1021/es902893f

[27] Amann RI, Ludwig W, Schleifer KH (1995) Phylogenetic Identification and in-situ detection of individual microbial cells without cultivation. Microbiol Rev 59: 143–169. 7535888 10.1128/mr.59.1.143-169.1995 PMC239358

[28] Allen HK, Moe LA, Rodbumrer J, Gaarder A, Handelsman J (2009) Functional metagenomics reveals diverse beta-lactamases in a remote Alaskan soil. ISME J 3: 243–251. 18843302 10.1038/ismej.2008.86

[29] Donato JJ, Moe LA, Converse BJ, Smart KD, Berklein FC, et al (2010) Metagenomic analysis of apple orchard soil reveals antibiotic resistance genes encoding predicted bifunctional proteins. Appl Environ Microbiol 76: 4396–4401. 20453147 10.1128/AEM.01763-09 PMC2897439

[30] Torres-Cortes G, Millan V, Ramirez-Saad HC, Nisa-Martinez R, Toro N, et al (2011) Characterization of novel antibiotic resistance genes identified by functional metagenomics on soil samples. Environ Microbiol 13: 1101–1114. 21281423 10.1111/j.1462-2920.2010.02422.x

[31] Szczepanowski R, Bekel T, Goesmann A, Krause L, Kromeke H, et al (2008) Insight into the plasmid metagenome of wastewater treatment plant bacteria showing reduced susceptibility to antimicrobial drugs analysed by the 454-pyrosequencing technology. J Biotechnol 136: 54–64. 18586057 10.1016/j.jbiotec.2008.03.020

[32] Kristiansson E, Fick J, Janzon A, Grabic R, Rutgersson C, et al (2011) Pyrosequencing of antibiotic-contaminated river sediments reveals high levels of resistance and gene transfer elements. PLoS One 6: e17038. 21359229 10.1371/journal.pone.0017038 PMC3040208

[33] Sommer MO, Dantas G, Church GM (2009) Functional characterization of the antibiotic resistance reservoir in the human microflora. Science 325: 1128–1131. 19713526 10.1126/science.1176950 PMC4720503

[34] Riesenfeld CS, Goodman RM, Handelsman J (2004) Uncultured soil bacteria are a reservoir of new antibiotic resistance genes. Environ Microbiol 6: 981–989. 15305923 10.1111/j.1462-2920.2004.00664.x

[35] Babson AL, Kawase A, MacCready P (2006) Seasonal and interannual variability in the circulation of Puget Sound, Washington: A box model study. Atmos Ocean 44: 29–45.

[36] Washington State Department of Ecology and Herrera Environmental Consultants, Inc. (2010) Phase 3: Loadings of toxic chemicals to Puget Sound from POTW Discharge of treated wastewater. Olympia. 241 p.

[37] Washington State Department of Health (2012) 2011 Annual Report: Commercial and recreational shellfish areas in Washington State. Olympia. 22 p.

[38] King County (2011) Wastewater treatment process: How is wastewater treated at King County’s West Point Treatment Plant? Available: http://www.kingcounty.gov/environment/wtd/About/System/West/Process.aspx. Accessed 22 February 2012.

[39] Meyer F, Paarmann D, D’Souza M, Olson R, Glass EM, et al (2008) The metagenomics RAST server - a public resource for the automatic phylogenetic and functional analysis of metagenomes. BMC Bioinformatics 9: 386. 18803844 10.1186/1471-2105-9-386 PMC2563014

[40] Huson DH, Auch AF, Qi J, Schuster SC (2007) MEGAN analysis of metagenomic data. Genome Res 17: 377–386. 17255551 10.1101/gr.5969107 PMC1800929

[41] Wang Q, Garrity GM, Tiedje JM, Cole JR (2007) Naive Bayesian classifier for rapid assignment of rRNA sequences into the new bacterial taxonomy. Appl Environ Microbiol 73: 5261–5267. 17586664 10.1128/AEM.00062-07 PMC1950982

[42] Rusch DB, Halpern AL, Sutton G, Heidelberg KB, Williamson S, et al (2007) The Sorcerer II Global Ocean Sampling expedition: northwest Atlantic through eastern tropical Pacific. PLoS Biol 5: e77. 17355176 10.1371/journal.pbio.0050077 PMC1821060

[43] Oh S, Caro-Quintero A, Tsementzi D, DeLeon-Rodriguez N, Luo C, et al (2011) Metagenomic insights into the evolution, function, and complexity of the planktonic microbial community of Lake Lanier, a temperate freshwater ecosystem. Appl Environ Microbiol 77: 6000–6011. 21764968 10.1128/AEM.00107-11 PMC3165412

[44] Tringe SG, von Mering C, Kobayashi A, Salamov AA, Chen K, et al (2005) Comparative metagenomics of microbial communities. Science 308: 554–557. 15845853 10.1126/science.1107851

[45] Liu B, Pop M (2009) ARDB-Antibiotic Resistance Genes Database. Nucleic Acids Res 37: D443–447. 18832362 10.1093/nar/gkn656 PMC2686595

[46] Zhu W, Lomsadze A, Borodovsky M (2010) Ab initio gene identification in metagenomic sequences. Nucleic Acids Res 38: e132. 20403810 10.1093/nar/gkq275 PMC2896542

[47] Camacho C, Coulouris G, Avagyan V, Ma N, Papadopoulos J, et al (2009) BLAST+: architecture and applications. BMC Bioinformatics 10: 421. 20003500 10.1186/1471-2105-10-421 PMC2803857

[48] Zhang T, Zhang XX, Ye L (2011) Plasmid metagenome reveals high levels of antibiotic resistance genes and mobile genetic elements in activated sludge. PLoS One 6: e26041. 22016806 10.1371/journal.pone.0026041 PMC3189950

[49] Punta M, Coggill PC, Eberhardt RY, Mistry J, Tate J, et al (2012) The Pfam protein families database. Nucleic Acids Res 40: D290–301. 22127870 10.1093/nar/gkr1065 PMC3245129

[50] Parks DH, Beiko RG (2010) Identifying biologically relevant differences between metagenomic communities. Bioinformatics 26: 715–721. 20130030 10.1093/bioinformatics/btq041

[51] Benjamini Y, Hochberg Y (1995) Controlling the false discovery rate: a practical and powerful approach to multiple testing. J Roy Stat Soc B Met 57: 289–300.

[52] McCullagh P, Nelder JA (1989) Generalized linear models. Boca Raton: Chapman & Hall/CRC. 532 p.

[53] Zinger L, Amaral-Zettler LA, Fuhrman JA, Horner-Devine MC, Huse SM, et al (2011) Global patterns of bacterial beta-diversity in seafloor and seawater ecosystems. PLoS One 6: e24570. 21931760 10.1371/journal.pone.0024570 PMC3169623

[54] Von Mering C, Hugenholtz P, Raes J, Tringe SG, Doerks T, et al (2007) Quantitative phylogenetic assessment of microbial communities in diverse environments. Science 315: 1126–1130. 17272687 10.1126/science.1133420

[55] Iverson V, Morris RM, Frazar CD, Berthiaume CT, Morales RL, et al (2012) Untangling genomes from metagenomes: Revealing an uncultured class of marine Euryarchaeota. Science 335: 587–590. 22301318 10.1126/science.1212665

[56] Ottesen EA, Marin R, Preston CM, Young CR, Ryan JP, et al (2011) Metatranscriptomic analysis of autonomously collected and preserved marine bacterioplankton. ISME J 5: 1881–1895. 21716310 10.1038/ismej.2011.70 PMC3223310

[57] Kelly KM, Chistoserdov AY (2001) Phylogenetic analysis of the succession of bacterial communities in the Great South Bay (Long Island). FEMS Microbiol Ecol 35: 85–95. 11248393 10.1111/j.1574-6941.2001.tb00791.x

[58] Turnbaugh PJ, Ley RE, Hamady M, Fraser-Liggett CM, Knight R, et al (2007) The human microbiome project. Nature 449: 804–810. 17943116 10.1038/nature06244 PMC3709439

[59] Cook KL, Rothrock MJ, Lovanh N, Sorrell JK, Loughrin JH (2010) Spatial and temporal changes in the microbial community in an anaerobic swine waste treatment lagoon. Anaerobe 16: 74–82. 19539043 10.1016/j.anaerobe.2009.06.003

[60] Liu C, Finegold SM, Song Y, Lawson PA (2008) Reclassification of Clostridium coccoides, Ruminococcus hansenii, Ruminococcus hydrogenotrophicus, Ruminococcus luti, Ruminococcus productus and Ruminococcus schinkii as Blautia coccoides gen. nov., comb. nov., Blautia hansenii comb. nov., Blautia hydrogenotrophica comb. nov., Blautia luti comb. nov., Blautia producta comb. nov., Blautia schinkii comb. nov. and description of Blautia wexlerae sp. nov., isolated from human faeces. Int J Syst Evol Microbiol 58: 1896–1902. 18676476 10.1099/ijs.0.65208-0

[61] Claesson MJ, O’Sullivan O, Wang Q, Nikkila J, Marchesi JR, et al (2009) Comparative analysis of pyrosequencing and a phylogenetic microarray for exploring microbial community structures in the human distal intestine. PLoS One 4: e6669. 19693277 10.1371/journal.pone.0006669 PMC2725325

[62] Newton RJ, Vandewalle JL, Borchardt MA, Gorelick MH, McLellan SL (2011) Lachnospiraceae and Bacteroidales alternative fecal indicators reveal chronic human sewage contamination in an urban harbor. Appl Environ Microbiol 77: 6972–6981. 21803887 10.1128/AEM.05480-11 PMC3187108

[63] McLellan SL, Huse SM, Mueller-Spitz SR, Andreishcheva EN, Sogin ML (2010) Diversity and population structure of sewage-derived microorganisms in wastewater treatment plant influent. Environ Microbiol 12: 378–392. 19840106 10.1111/j.1462-2920.2009.02075.x PMC2868101

[64] Albertsen M, Hansen LB, Saunders AM, Nielsen PH, Nielsen KL (2012) A metagenome of a full-scale microbial community carrying out enhanced biological phosphorus removal. ISME J 6: 1094–1106. 22170425 10.1038/ismej.2011.176 PMC3358022

[65] Lozupone CA, Knight R (2007) Global patterns in bacterial diversity. Proc Natl Acad Sci USA 104: 11436–11440. 17592124 10.1073/pnas.0611525104 PMC2040916

[66] Gilbert JA, Steele JA, Caporaso JG, Steinbrueck L, Reeder J, et al (2012) Defining seasonal marine microbial community dynamics. ISME J 6: 298–308. 21850055 10.1038/ismej.2011.107 PMC3260500

[67] Gilbert JA, Dupont CL (2011) Microbial metagenomics: beyond the genome. Ann Rev Mar Sci 3: 347–371. 10.1146/annurev-marine-120709-142811 21329209

[68] Munir M, Wong K, Xagoraraki I (2011) Release of antibiotic resistant bacteria and genes in the effluent and biosolids of five wastewater utilities in Michigan. Water Res 45: 681–693. 20850863 10.1016/j.watres.2010.08.033

[69] Zhang Y, Marrs CF, Simon C, Xi C (2009) Wastewater treatment contributes to selective increase of antibiotic resistance among Acinetobacter spp. Sci Total Environ 407: 3702–3706. 19321192 10.1016/j.scitotenv.2009.02.013

[70] Ferreira da Silva M, Tiago I, Verissimo A, Boaventura RA, Nunes OC, et al (2006) Antibiotic resistance of enterococci and related bacteria in an urban wastewater treatment plant. FEMS Microbiol Ecol 55: 322–329. 16420639 10.1111/j.1574-6941.2005.00032.x

[71] Uyaguari MI, Fichot EB, Scott GI, Norman RS (2011) Characterization and quantitation of a novel beta-lactamase gene found in a wastewater treatment facility and the surrounding coastal ecosystem. Appl Environ Microbiol 77: 8226–8233. 21965412 10.1128/AEM.02732-10 PMC3233035

[72] Boerlin P, Reid-Smith RJ (2008) Antimicrobial resistance: its emergence and transmission. Anim Health Res Rev 9: 115–126. 19102787 10.1017/S146625230800159X

[73] Partridge SR, Tsafnat G, Coiera E, Iredell JR (2009) Gene cassettes and cassette arrays in mobile resistance integrons. FEMS Microbiol Rev 33: 757–784. 19416365 10.1111/j.1574-6976.2009.00175.x

[74] Moran MA, Buchan A, Gonzalez JM, Heidelberg JF, Whitman WB, et al (2004) Genome sequence of Silicibacter pomeroyi reveals adaptations to the marine environment. Nature 432: 910–913. 15602564 10.1038/nature03170

[75] Furushita M, Akagi H, Kaneoka A, Awamura K, Maeda T, et al (2011) Structural variation of Tn10 that carries tetB found in fish farm bacteria. Microbes Environ 26: 84–87. 21487208 10.1264/jsme2.me10160

[76] Schluter A, Krahn I, Kollin F, Bonemann G, Stiens M, et al (2007) IncP-1-beta plasmid pGNB1 isolated from a bacterial community from a wastewater treatment plant mediates decolorization of triphenylmethane dyes. Appl Environ Microbiol 73: 6345–6350. 17675426 10.1128/AEM.01177-07 PMC2075058

[77] Suenaga H, Koyama Y, Miyakoshi M, Miyazaki R, Yano H, et al (2009) Novel organization of aromatic degradation pathway genes in a microbial community as revealed by metagenomic analysis. ISME J 3: 1335–1348. 19587775 10.1038/ismej.2009.76

[78] Jencova V, Strnad H, Chodora Z, Ulbrich P, Vlcek C, et al (2008) Nucleotide sequence, organization and characterization of the (halo)aromatic acid catabolic plasmid pA81 from Achromobacter xylosoxidans A8. Res Microbiol 159: 118–127. 18249097 10.1016/j.resmic.2007.11.018

[79] Monchy S, Benotmane MA, Janssen P, Vallaeys T, Taghavi S, et al (2007) Plasmids pMOL28 and pMOL30 of Cupriavidus metallidurans are specialized in the maximal viable response to heavy metals. J Bacteriol 189: 7417–7425. 17675385 10.1128/JB.00375-07 PMC2168447

[80] Sajjaphan K, Shapir N, Wackett LP, Palmer M, Blackmon B, et al (2004) Arthrobacter aurescens TC1 atrazine catabolism genes trzN, atzB, and atzC are linked on a 160-kilobase region and are functional in Escherichia coli. Appl Environ Microbiol 70: 4402–4407. 15240330 10.1128/AEM.70.7.4402-4407.2004 PMC444770

[81] DeLong EF, Preston CM, Mincer T, Rich V, Hallam SJ, et al (2006) Community genomics among stratified microbial assemblages in the ocean’s interior. Science 311: 496–503. 16439655 10.1126/science.1120250

[82] Zeigler Allen L, Allen EE, Badger JH, McCrow JP, Paulsen IT, et al (2012) Influence of nutrients and currents on the genomic composition of microbes across an upwelling mosaic. ISME J 6: 1403–1414. 22278668 10.1038/ismej.2011.201 PMC3379637

