# Supplementary figures and images for: Metagenomic Profiling of Microbial Composition and Antibiotic Resistance Determinants in Puget Sound
Source: PLoS One. 2012 Oct 29;7(10):e48000. doi: 10.1371/journal.pone.0048000 (PMC3483302; doi:10.1371/journal.pone.0048000)

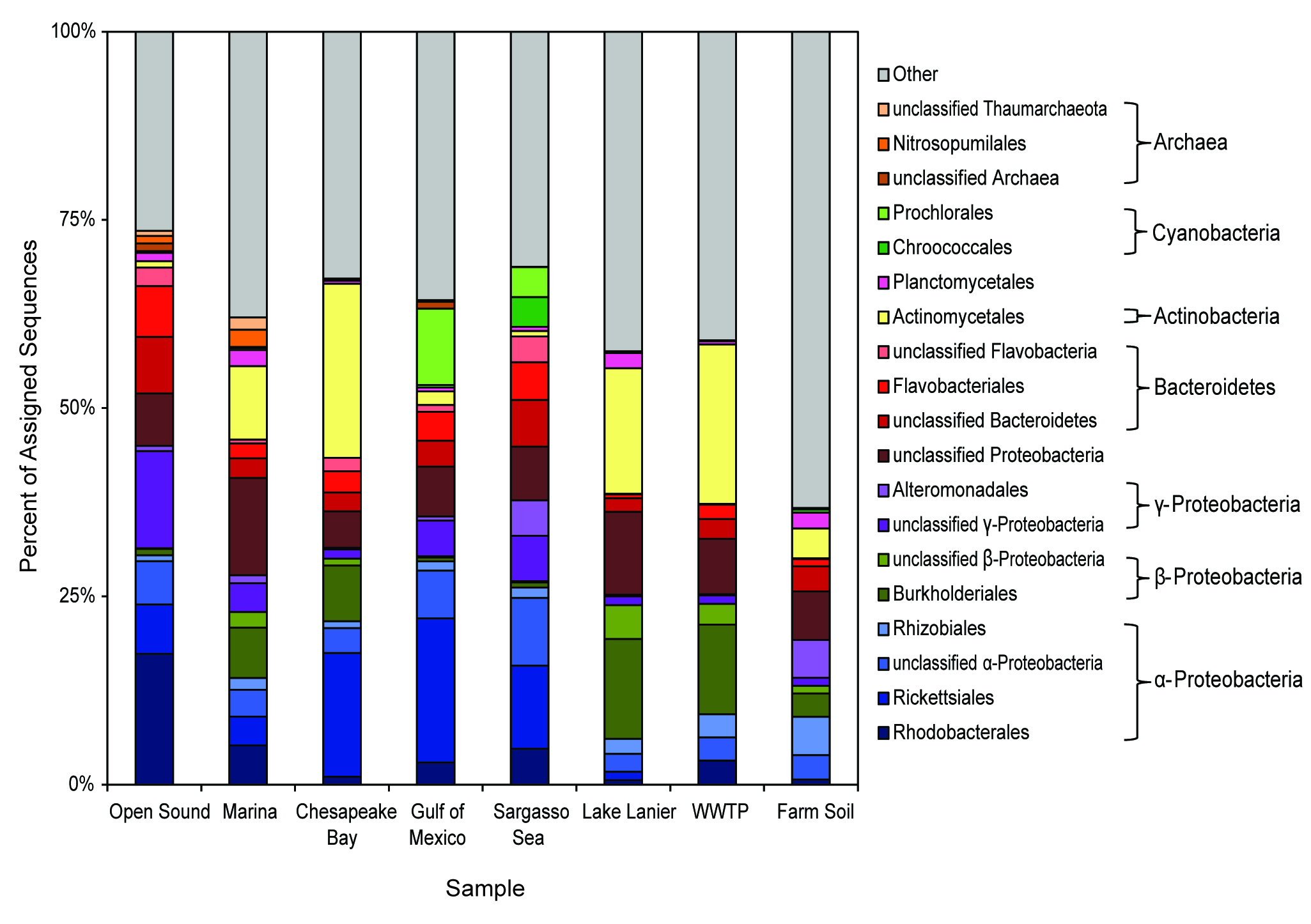

Supplement: Figure S1 — Relative abundance (order level) of major taxonomic groups in the Puget Sound samples and other selected metagenomes. Sequences were assigned to the NCBI taxonomy using MG-Rast [39] and the lowest common ancestor algorithm (≥50% identity and alignment length ≥50 amino acids). Taxa representing >1% of assignable sequences in one or more samples are shown, while taxa present in <1% of sequences in all samples are grouped in the ‘other’ category. (TIF) [file pone.0048000.s001.tif]

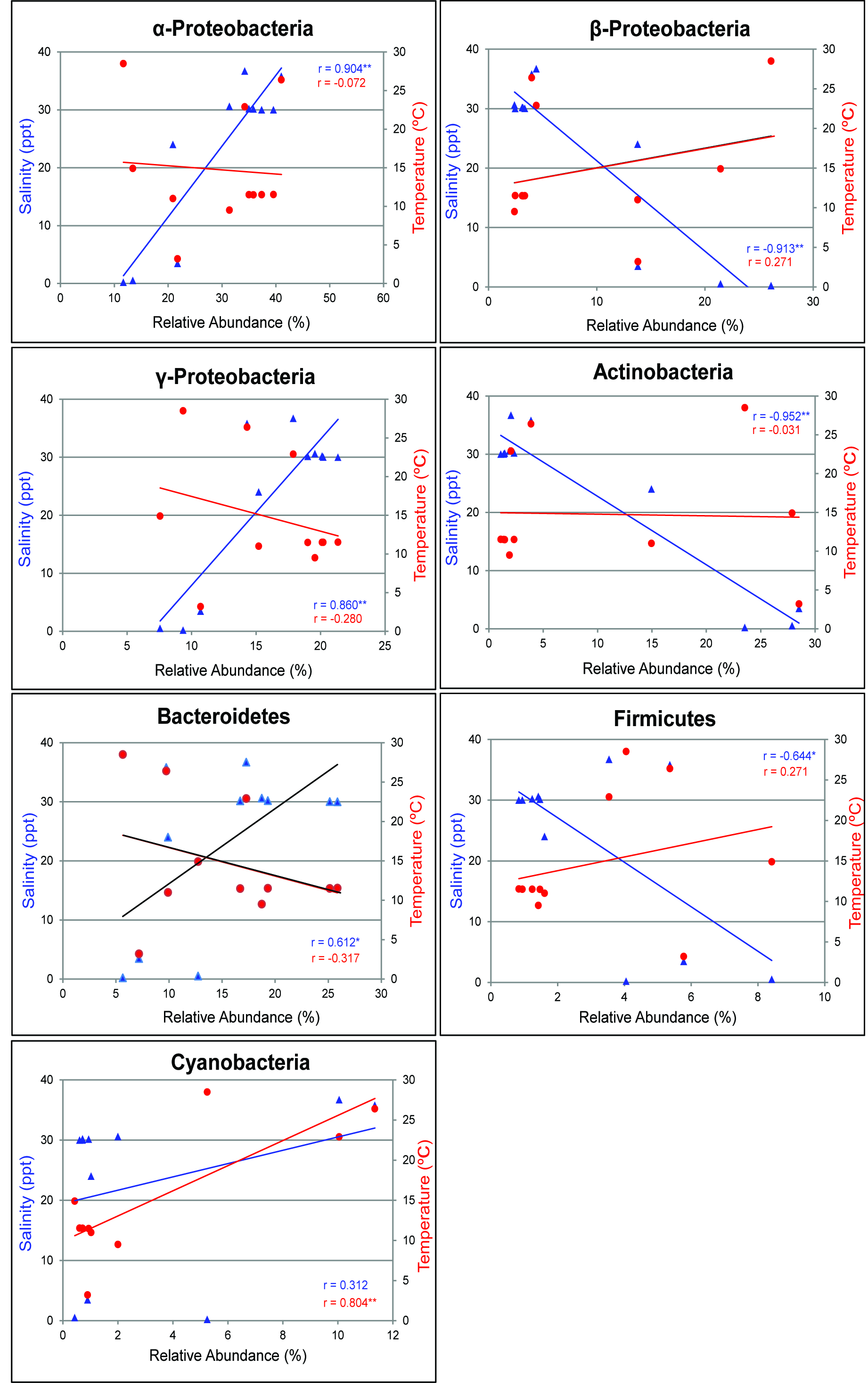

Supplement: Figure S2 — Relationship between the relative abundance of predominant taxa and salinity and temperature gradients. Linear regression lines and Pearson’s coefficients (*p<0.05 and **p<0.005) are shown. Data points include the open Sound, Marina, WWTP, Chesapeake Bay, Sargasso Sea, Gulf of Mexico and Lake Lanier metagenomic samples. (TIF) [file pone.0048000.s002.tif]

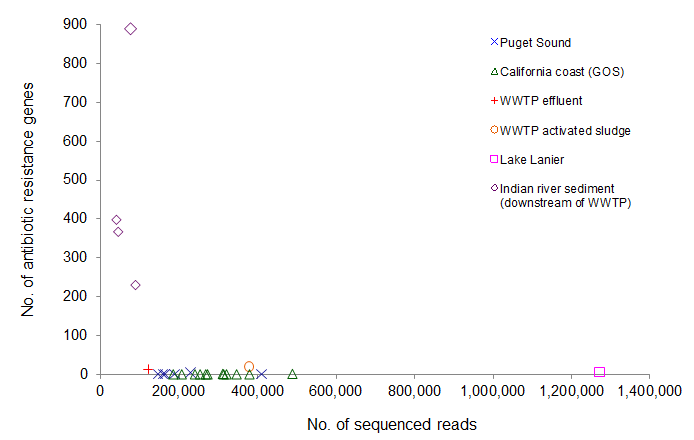

Supplement: Figure S3 — Abundance of antibiotic resistance gene sequences in environmental metagenomes with varying sequencing depth. These metagenomes represent a diverse mix of environments, including river sediment samples taken downstream from a wastewater treatment plant (WWTP) processing high volumes of antibiotics [32], coastal surface water samples taken as part of the Global Ocean Sampling Expedition [82], the activated sludge fraction of a WWTP [15] and an urban freshwater lake [43]. Reads that aligned to a sequence within the ARDB+ with ≥80% sequence identity over at least 50 amino acids were classified as putative resistance genes. (TIF) [file pone.0048000.s003.tif]
