# Supplementary material for: Metagenomic Profiling of Microbial Composition and Antibiotic Resistance Determinants in Puget Sound
Source: PLoS One. 2012 Oct 29;7(10):e48000. doi: 10.1371/journal.pone.0048000 (PMC3483302; doi:10.1371/journal.pone.0048000)
Supplement: Table S1 — Puget Sound 454 sequence reads with ≥80% similarity and an alignment length ≥50 amino acids to sequences within the expanded Antibiotic Resistance Genes Database (ARDB+). (PDF) [file pone.0048000.s004.pdf]

Table S1. Puget Sound 454 sequence reads with ≥80% similarity and an alignment length ≥50 amino acids to sequences within the expanded Antibiotic Resistance Genes Database (ARDB+).

454 Read ID	Sample	Best hit to ARDB+	Percent identity	Alignment length (aa)	Query coverage	Antibiotic resistance gene	Antibiotic resistance class	Species	Best hit Genbank ID	Genbank protein
GYS1QVW02GFSBG	WWTP	122934824	96	141	57	BL3_imp	Beta-lactam	*Serratia marcescens*	122934824	Metallo-beta-lactamase
GYS1QVW02JR7KA	WWTP	58200478	100	120	56	tetA(39)	Tetracycline	*Acinetobacter sp. LUH5605*	58200478	TetR(39)
GYS1QVW01EZL9G	Marina	241992557	100	53	55	qacG2	Quaternary ammonium compounds	*Uncultured bacterium*	241992557	QacG
GYS1QVW01C3WDM	WWTP	314993276	95	137	55	ermB	Macrolide	*Enterococcus faecium TX0133B*	256964193	rRNA methylase
GYS1QVW01EPBXP	WWTP	314993276	100	132	53	ermB	Macrolide	*Enterococcus faecium TX0133B*	146285381	Ribosomal methylase
GYS1QVW01A42OG	WWTP	289812760	89	119	51	BL2d_ oxa10	Beta-lactam	*Providencia rettgeri*	56791710	OXA-10 beta lactamase
GYS1QVW02IDABT	WWTP	224496169	99	144	42	MefA	Macrolide	*Streptococcus anginosus*	169809207	Macrolide-efflux protein
GYS1QVW02G6714	WWTP	58200479	94	155	39	tet39	Tetracycline	*Acinetobacter sp. LUH5605*	58200479	TetA(39)
GYS1QVW02GVJSG	Marina	121604263	82	108	39	BacA	Bacitracin	*Polaromonas naphthalenivorans CJ2*	332528682	UDP pyrophospate phosphatase
GYS1QVW02HMRHR	Marina	84616919	80	151	38	orf11	Beta-lactam	*Desulfococcus multivorans*	84616919	Putative ABC-type permease
GYS1QVW02JRJX1	WWTP	283798930	99	132	33	tet40	Tetracycline	*Clostridium sp. M62/1*	283798930	MDR-type permease, tet resistance protein
GYS1QVW02F32RH	WWTP	296285266	99	161	25	tetW	Tetracycline	*Corynebacterium resistens*	323141252	Elongation factor G
GYS1QVW01AG7BR	WWTP	56479010	85	74	19	orf11	Beta-lactam	*Aromatoleum aromaticum EbN1*	56479010	ABC transporter permease
GYS1QVW01DHHKX	Marina	146306240	83	141	14	MexW	Multidrug resistant efflux protein	*Pseudomonas mendocina ymp*	77460714	Multidrug efflux protein
GYS1QVW02HM984	WWTP	254967143	97	66	10	tetW	Tetracycline	*Uncultured organism*	204789616	Tetracycline resistance protein
GYS1QVW02FI0S3	WWTP	254967143	100	50	8	tetW	Tetracycline	*Uncultured organism*	204789616	Tetracycline resistance protein
GYS1QVW02JXAIU	WWTP	260597281	82	50	8	MacB	Macrolide	*Cronobacter turicensis z3032*	346224427	Hypothetical protein
GYS1QVW02JY5OX	WWTP	260596113	87	62	6	AcrB	Macrolide	*Cronobacter turicensis z3032*	320540972	putative multidrug efflux system protein
