# Supplementary material for: Metagenomic Profiling of Microbial Composition and Antibiotic Resistance Determinants in Puget Sound
Source: PLoS One. 2012 Oct 29;7(10):e48000. doi: 10.1371/journal.pone.0048000 (PMC3483302; doi:10.1371/journal.pone.0048000)
Supplement: Table S2 — Plasmid sequences in the NCBI RefSeq database that match contigs from the Puget Sound and wastewater treatment plant (WWTP) effluent metagenomic datasets. (PDF) [file pone.0048000.s005.pdf]

Table S2. Plasmid sequences in the NCBI RefSeq database that match contigs from the Puget Sound and wastewater treatment plant (WWTP) effluent metagenomic datasets.

Contig ID	Location	Plasmid name	GI number	Bacterial host	Identity (%)	Hit length (bp)
00012	WWTP	pGNB1	149350899	*Uncultured bacterium*	100	2,130
00060	WWTP	pSKYE1	255292227	*Uncultured bacterium*	100	1,127
00059	WWTP	pAOVO01	120608524	*Acidovorax sp.*	100	1,093
01935	P5	TM1040	99034845	*Silicibacter sp.*	97	537
00155	WWTP	pK214	2467210	*Lactococcus lactis*	97	749
00139	P32	TM1040	99034845	*Silicibacter sp.*	96	562
00332	P1	TM1040	99034845	*Silicibacter sp.*	96	556
