# Supplementary material for: Metagenomic Profiling of Microbial Composition and Antibiotic Resistance Determinants in Puget Sound
Source: PLoS One. 2012 Oct 29;7(10):e48000. doi: 10.1371/journal.pone.0048000 (PMC3483302; doi:10.1371/journal.pone.0048000)
Supplement: Table S3 — Plasmid sequences in the NCBI RefSeq database that match sequence reads from the Puget Sound and wastewater treatment plant (WWTP) effluent datasets. (PDF) [file pone.0048000.s006.pdf]

**Table S3.** Plasmid sequences in the NCBI RefSeq database that match sequence reads from the Puget Sound and wastewater treatment plant (WWTP) effluent datasets.

| GI number | Plasmid name | Bacterial host | Identity (%) ≥ | Hit length (bp) ≥ | No. of reads | Location |
|---|---|---|---|---|---|---|
| 99034845 | unnamed | *Silicibacter sp.* | 95 | 109 | 60 | P1, P5, P26, P28, P32, Marina |
| 56410263 | pMOL28 | *Ralstonia metallidurans* | 97 | 102 | 38 | P28, WWTP |
| 310639221 | pYP1 | *Ketogulonicigenium vulgare* | 95 | 108 | 29 | P1, P5, P26, P28, P32, Marina, WWTP |
| 32263857 | pOL18 | *Paracoccus sp.* | 95 | 106 | 19 | P1, P5, P26, P32, Marina |
| 299068436 | CMR15_mp | *Ralstonia solanacearum* | 95 | 116 | 13 | P5, Marina, WWTP |
| 255292227 | pSKYE1 | *Uncultured bacterium* | 96 | 261 | 13 | WWTP |
| 51470556 | pFBAOT6 | *Aeromonas punctata* | 97 | 238 | 11 | P32, WWTP |
| 221236817 | unnamed | *Escherichia sp.* | 95 | 105 | 9 | P1, P5, P26, P28, P32, Marina, WWTP |
| 145557411 | pRSPA01 | *Rhodobacter sphaeroides* | 95 | 128 | 9 | P1, P5, Marina, WWTP |
| 16605595 | pRVS1 | *Vibrio salmonicida* | 98 | 241 | 9 | Marina |
| 33867057 | pBD2 | *Rhodococcus erythropolis* | 98 | 300 | 8 | WWTP |
| 326407899 | pCV56A | *Lactococcus lactis* | 98 | 259 | 7 | WWTP |
| 146322225 | pGdh442 | *Lactococcus lactis* | 96 | 266 | 7 | WWTP |
| 66862639 | Rms149 | *Pseudomonas aeruginosa* | 99 | 178 | 7 | Marina, WWTP |
| 2467210 | pK214 | *Lactococcus lactis* | 96 | 128 | 6 | WWTP |
| 18077099 | pWW0 | *Pseudomonas putida* | 100 | 119 | 6 | WWTP |
| 113473678 | pCAR3 | *Sphingomonas sp.* | 96 | 251 | 6 | WWTP |
| 149350899 | pGNB1 | *Uncultured bacterium* | 97 | 225 | 6 | WWTP |
| 120608524 | pAOVO01 | *Acidovorax sp.* | 98 | 264 | 5 | WWTP |
| 76151975 | pA17sv1 | *Enterococcus faecium* | 97 | 126 | 5 | WWTP |
| 238873439 | unnamed | *Eubacterium eligens* | 98 | 217 | 5 | WWTP |
| 326407968 | pCV56C | *Lactococcus lactis* | 98 | 226 | 5 | WWTP |
| 24394861 | pKLH201 | *Acinetobacter calcoaceticus* | 99 | 239 | 4 | WWTP |
| 288959735 | pAB510a | *Azospirillum sp.* | 95 | 170 | 4 | P1, P5, P32, Marina |
| 306415518 | pMC1 | *Delftia acidovorans* | 96 | 193 | 4 | WWTP |

| | | | | | | |
|---|---|---|---|---|---|---|
| 239976803 | unnamed | *Enterococcus faecium* | 99 | 320 | 4 | WWTP |
| 179348463 | pMPOP01 | *Methylobacterium populi* | 99 | 230 | 4 | WWTP |
| 321170327 | pRAHAQ01 | *Rahnella sp.* | 99 | 251 | 4 | P5, Marina |
| 194709275 | pCVM19633_110 | *Salmonella enterica* | 100 | 158 | 4 | Marina |
| 292677436 | pCHQ1 | *Sphingobium japonicum* | 95 | 322 | 4 | WWTP |
| 31746361 | pB10 | *Uncultured bacterium* | 99 | 187 | 4 | WWTP |
| 58416215 | pA81 | *Achromobacter xylosoxidans* | 99 | 148 | 3 | WWTP |
| 283484478 | pMMD | *Acinetobacter baumannii* | 97 | 315 | 3 | P32, WWTP |
| 83833713 | pAV2 | *Acinetobacter venetianus* | 98 | 329 | 3 | P28, WWTP |
| 307111956 | pBUN24 | *Bacteroides uniformis* | 98 | 191 | 3 | WWTP |
| 30387438 | PNAC2 | *Bifidobacterium longum* | 99 | 362 | 3 | WWTP |
| 57019077 | pCC178 | *Campylobacter coli* | 97 | 226 | 3 | WWTP |
| 17059596 | pTET3 | *Corynebacterium glutamicum* | 98 | 179 | 3 | WWTP |
| 34500478 | pUO1 | *Delftia acidovorans* | 98 | 165 | 3 | WWTP |
| 121490884 | pVEF1 | *Enterococcus faecium* | 98 | 159 | 3 | WWTP |
| 38016624 | pLVPK | *Klebsiella pneumoniae* | 95 | 340 | 3 | Marina |
| 150958389 | pKPN4 | *Klebsiella pneumoniae* | 95 | 185 | 3 | WWTP |
| 179366399 | pLR581 | *Lactobacillus reuteri* | 98 | 325 | 3 | WWTP |
| 183178785 | pMM23 | *Mycobacterium marinum* | 99 | 453 | 3 | WWTP |
| 17548221 | pGMI1000MP | *Ralstonia solanacearum* | 95 | 227 | 3 | Marina, WWTP |
| 160431608 | pMAK2 | *Salmonella enterica* | 95 | 273 | 3 | WWTP |
| 295797733 | pTINT01 | *Thiomonas intermedia* | 95 | 257 | 3 | P1, WWTP |
| 205320843 | pHHV35 | *Uncultured bacterium* | 96 | 167 | 3 | WWTP |
| 311109684 | pA81 | *Achromobacter xylosoxidans* | 99 | 314 | 2 | WWTP |
| 58200477 | ptet5605 | *Acinetobacter sp.* | 100 | 432 | 2 | WWTP |
| 30172175 | pKLH205 | *Acinetobacter sp.* | 99 | 178 | 2 | WWTP |
| 10957030 | pRAY | *Acinetobacter sp.* | 95 | 115 | 2 | WWTP |
| 294351974 | pBM400 | *Bacillus megaterium* | 95 | 199 | 2 | WWTP |

| | | | | | | |
|---|---|---|---|---|---|---|
| 194359988 | pBFP35 | *Bacteroides fragilis* | 99 | 419 | 2 | WWTP |
| 146411246 | pBBta01 | *Bradyrhizobium sp.* | 96 | 159 | 2 | Marina |
| 134132180 | pBVIE02 | *Burkholderia vietnamiensis* | 100 | 250 | 2 | WWTP |
| 57019266 | pCC178 | *Campylobacter coli* | 99 | 385 | 2 | WWTP |
| 190571920 | pCNB | *Comamonas testosteroni* | 99 | 102 | 2 | WWTP |
| 295059951 | pECL_A | *Enterobacter cloacae* | 99 | 403 | 2 | Marina, WWTP |
| 12956985 | pRE25 | *Enterococcus faecalis* | 99 | 261 | 2 | WWTP |
| 309385929 | pLG1 | *Enterococcus faecium* | 97 | 437 | 2 | WWTP |
| 241248270 | unnamed | *Enterococcus faecium* | 95 | 394 | 2 | WWTP |
| 239977009 | unnamed | *Enterococcus faecium* | 98 | 242 | 2 | WWTP |
| 193783420 | pJIBE401 | *Klebsiella pneumoniae* | 98 | 161 | 2 | Marina |
| 171854418 | pKL0018 | *Lactococcus garvieae* | 99 | 119 | 2 | WWTP |
| 326407939 | pCV56B | *Lactococcus lactis* | 96 | 342 | 2 | WWTP |
| 190571754 | pNP40 | *Lactococcus lactis* | 99 | 284 | 2 | WWTP |
| 116108915 | Plasmid 3 | *Lactococcus lactis* | 97 | 245 | 2 | WWTP |
| 44078 | lactose | *Lactococcus lactis* | 96 | 179 | 2 | WWTP |
| 32455473 | pAH82 | *Lactococcus lactis* | 98 | 478 | 2 | WWTP |
| 47018986 | pLM80 | *Listeria monocytogenes* | 98 | 235 | 2 | WWTP |
| 32469878 | pDTG1 | *Pseudomonas putida* | 95 | 384 | 2 | WWTP |
| 190572013 | pCT14 | *Pseudomonas sp.* | 98 | 180 | 2 | Marina, WWTP |
| 56068618 | pMOL30 | *Ralstonia metallidurans* | 96 | 309 | 2 | WWTP |
| 77454567 | pREL1 | *Rhodococcus erythropolis* | 99 | 446 | 2 | WWTP |
| 148550551 | pSWIT01 | *Sphingomonas wittichii* | 99 | 376 | 2 | WWTP |
| 317109770 | PB5 | *Uncultured bacterium* | 98 | 106 | 2 | WWTP |
| 57236769 | pKA1 | *Vibrio cholerae* | 96 | 482 | 2 | Marina |
| 183211582 | pACICU1 | *Acinetobacter baumannii* | 100 | 145 | 1 | Marina |
| 30409103 | pKLH207 | *Actinobacter sp.* | 99 | 286 | 1 | WWTP |
| 142855988 | Plasmid 4 | *Aeromonas salmonicida* | 100 | 433 | 1 | WWTP |

| | | | | | | |
|---|---|---|---|---|---|---|
| 317119630 | pALIDE01 | *Alicycliphilus denitrificans* | 99 | 368 | 1 | WWTP |
| 288960867 | pAB510b | *Azospirillum sp.* | 96 | 212 | 1 | P1 |
| 288962595 | pAB510e | *Azospirillum sp.* | 95 | 129 | 1 | Marina |
| 222822840 | unnamed | *Bacteroides fragilis* | 100 | 418 | 1 | WWTP |
| 222822802 | unnamed | *Bacteroides sp.* | 100 | 431 | 1 | WWTP |
| 222822750 | unnamed | *Bacteroides sp.* | 96 | 307 | 1 | WWTP |
| 853781 | pIP417 | *Bacteroides sp.* | 100 | 413 | 1 | WWTP |
| 73665544 | pBIF10 | *Bifidobacterium bifidum* | 100 | 289 | 1 | WWTP |
| 121505102 | pTet | *Campylobacter jejuni* | 100 | 358 | 1 | WWTP |
| 57118012 | pCG8245 | *Campylobacter jejuni* | 99 | 328 | 1 | WWTP |
| 257048712 | pAph03 | *Candidatus Accumulibacter* | 96 | 451 | 1 | WWTP |
| 50727963 | pTSA | *Comamonas testosteroni* | 98 | 359 | 1 | WWTP |
| 296172990 | pJA144188 | *Corynebacterium resistens* | 96 | 153 | 1 | WWTP |
| 89513168 | pLEW279a | *Corynebacterium sp.* | 98 | 169 | 1 | WWTP |
| 32479367 | pTP10 | *Corynebacterium striatum* | 99 | 512 | 1 | WWTP |
| 260600006 | pCTU3 | *Cronobacter turicensis* | 97 | 393 | 1 | Marina |
| 226319394 | Plasmid 1 | *Deinococcus deserti* | 95 | 116 | 1 | Marina |
| 113706807 | pDGEO01 | *Deinococcus geothermalis* | 95 | 154 | 1 | P26 |
| 226807567 | pEC-IMP | *Enterobacter cloacae* | 97 | 426 | 1 | WWTP |
| 270208272 | pBEE99 | *Enterococcus faecalis* | 99 | 221 | 1 | WWTP |
| 241252865 | unnamed | *Enterococcus faecalis* | 100 | 205 | 1 | WWTP |
| 239825540 | unnamed | *Enterococcus faecalis* | 99 | 186 | 1 | WWTP |
| 190350257 | pVEF3 | *Enterococcus faecium* | 100 | 459 | 1 | WWTP |
| 172051323 | pIP1206 | *Escherichia coli* | 99 | 424 | 1 | WWTP |
| 99867038 | pAPEC-O1-R | *Escherichia coli* | 100 | 366 | 1 | WWTP |
| 89513164 | pLEW517 | *Escherichia coli* | 100 | 176 | 1 | WWTP |
| 89033265 | NR1 | *Escherichia coli* | 100 | 275 | 1 | WWTP |
| 12024948 | R751 | *Escherichia coli* | 99 | 361 | 1 | WWTP |

| | | | | | | |
|---|---|---|---|---|---|---|
| 195537732 | pLTK13 | *Lactobacillus plantarum* | 99 | 369 | 1 | P5 |
| 32455506 | pMD5057 | *Lactobacillus plantarum* | 99 | 200 | 1 | Marina |
| 257152781 | N/A | *Lactobacillus rhamnosus* | 99 | 206 | 1 | WWTP |
| 125631981 | pEps352 | *Lactococcus lactis* | 98 | 150 | 1 | WWTP |
| 76574874 | pSK11L | *Lactococcus lactis* | 99 | 427 | 1 | WWTP |
| 32455464 | pBL1 | *Lactococcus lactis* | 100 | 273 | 1 | WWTP |
| 9507248 | pND861 | *Lactococcus lactis* | 98 | 524 | 1 | WWTP |
| 6739582 | pCI2000 | *Lactococcus lactis* | 95 | 462 | 1 | WWTP |
| 53755675 | pLPP | *Legionella pneumophila* | 97 | 241 | 1 | WWTP |
| 295831620 | LkipL4726 | *Leuconostoc kimchii* | 100 | 431 | 1 | WWTP |
| 222121345 | pMCCL2 | *Macrococcus caseolyticus* | 99 | 393 | 1 | WWTP |
| 82619190 | unnamed | *Mesorhizobium sp.* | 95 | 212 | 1 | P1 |
| 110346917 | Plasmid 1 | *Mesorhizobium sp.* | 100 | 317 | 1 | WWTP |
| 170658659 | pMRAD01 | *Methylobacterium radiotolerans* | 95 | 254 | 1 | P1 |
| 216774 | unnamed | *Moraxella sp.* | 99 | 378 | 1 | WWTP |
| 315265130 | pMSPYR101 | *Mycobacterium sp.* | 97 | 104 | 1 | WWTP |
| 325983714 | pNAL21201 | *Nitrosomonas sp.* | 99 | 489 | 1 | WWTP |
| 325980815 | pNAL21202 | *Nitrosomonas sp.* | 97 | 139 | 1 | WWTP |
| 145322134 | pNL1 | *Novosphingobium aromaticivorans* | 97 | 262 | 1 | WWTP |
| 294869143 | pAMI7 | *Paracoccus aminophilus* | 99 | 449 | 1 | WWTP |
| 154818276 | pMTH1 | *Paracoccus methylutens* | 100 | 379 | 1 | WWTP |
| 118504932 | pPRO2 | *Pelobacter propionicus* | 100 | 407 | 1 | WWTP |
| 121583017 | pPNAP02 | *Polaromonas naphthalenivorans* | 95 | 423 | 1 | WWTP |
| 120595973 | pPNAP01 | *Polaromonas naphthalenivorans* | 98 | 102 | 1 | WWTP |
| 194359396 | pOZ176 | *Pseudomonas aeruginosa* | 99 | 385 | 1 | P28 |
| 156104616 | pMATVIM-7 | *Pseudomonas aeruginosa* | 100 | 362 | 1 | WWTP |
| 599573 | pSA1700 | *Pseudomonas aeruginosa* | 95 | 487 | 1 | WWTP |
| 49188490 | pRA2 | *Pseudomonas alcaligenes* | 95 | 253 | 1 | WWTP |

| | | | | | | |
|---|---|---|---|---|---|---|
| 296100168 | pDK1 | *Pseudomonas putida* | 100 | 404 | 1 | Marina |
| 90576544 | NAH7 | *Pseudomonas putida* | 95 | 230 | 1 | WWTP |
| 42632299 | pND6-1 | *Pseudomonas sp.* | 100 | 437 | 1 | WWTP |
| 32455785 | pADP-1 | *Pseudomonas sp.* | 100 | 501 | 1 | WWTP |
| 299073288 | RCFBPv3_mp | *Ralstonia solanacearum* | 95 | 201 | 1 | WWTP |
| 77019866 | pREC1 | *Rhodococcus erythropolis* | 99 | 348 | 1 | WWTP |
| 145226750 | pDK2 | *Rhodococcus sp.* | 97 | 483 | 1 | WWTP |
| 456362 | pBT233 | *S.pyogenes* | 100 | 396 | 1 | WWTP |
| 18873674 | pUR400 | *S.typhimurium* | 100 | 243 | 1 | WWTP |
| 10957190 | R27 | *Salmonella typhi* | 100 | 246 | 1 | WWTP |
| 38259307 | R478 | *Serratia marcescens* | 99 | 301 | 1 | P32 |
| 117676079 | Plasmid 1 | *Shewanella sp.* | 99 | 342 | 1 | WWTP |
| 18462515 | pCP301 | *Shigella flexneri* | 100 | 105 | 1 | WWTP |
| 99035147 | unnamed | *Silicibacter sp.* | 96 | 466 | 1 | P1 |
| 292677706 | pUT2 | *Sphingobium japonicum* | 100 | 242 | 1 | WWTP |
| 110346757 | pYAN-1 | *Sphingobium yanoikuyae* | 95 | 287 | 1 | WWTP |
| 291167465 | pISP3 | *Sphingomonas sp.* | 100 | 350 | 1 | WWTP |
| 148550845 | pSWIT02 | *Sphingomonas wittichii* | 95 | 421 | 1 | WWTP |
| 284005967 | pSLIN06 | *Spirosoma linguale* | 96 | 141 | 1 | WWTP |
| 284005577 | pSLIN01 | *Spirosoma linguale* | 97 | 277 | 1 | WWTP |
| 85057109 | pMTSm1 | *Stenotrophomonas maltophilia* | 100 | 478 | 1 | WWTP |
| 251819044 | pBM407 | *Streptococcus suis* | 95 | 313 | 1 | WWTP |
| 10956194 | pER35 | *Streptococcus thermophilus* | 98 | 438 | 1 | WWTP |
| 54969619 | pRSB101 | *Uncultured bacterium* | 100 | 314 | 1 | Marina |
| 317109853 | PB11 | *Uncultured bacterium* | 95 | 200 | 1 | WWTP |
| 290791046 | pAKD4 | *Uncultured bacterium* | 100 | 384 | 1 | WWTP |
| 205320734 | pHHV216 | *Uncultured bacterium* | 100 | 388 | 1 | WWTP |
| 108859310 | pTRACA | *Uncultured bacterium* | 98 | 439 | 1 | WWTP |

| | | | | | | | |
|---|---|---|---|---|---|---|---|
| 84094946 | pTP6 | *Uncultured bacterium* | 100 | 427 | 1 | WWTP |
| 28875495 | pAK107 | *Uncultured bacterium* | 98 | 281 | 1 | WWTP |
| 19070006 | pB4 | *Uncultured bacterium* | 95 | 457 | 1 | WWTP |
| 119416936 | TC68 | *Vibrio sp.* | 100 | 453 | 1 | Marina |
| 154162790 | pXAUT01 | *Xanthobacter autotrophicus* | 100 | 491 | 1 | WWTP |
